# Dynamic stability analysis method of anchored rocky slope considering seismic deterioration effect

**DOI:** 10.1038/s41598-024-57413-3

**Published:** 2024-03-25

**Authors:** Jinqing Jia, Xing Gao, Xiaohua Bao, Xin Xiang, Lihua Zhang, Bingxiong Tu

**Affiliations:** 1 State Key Laboratory of Coastal and Offshore Engineering, School of Civil Engineering, Dalian University of Technology, Dalian, 116024 China; 2 College of Civil and Transportation Engineering, Shenzhen University, Shenzhen, 518060 China; 3 China Three Gorges Corporation, Wuhan, 430010 China; 4 Fujian Engineering Technology Research Center for Tunnel and Underground Space, Huaqiao University, Xiamen, 361021 China

**Keywords:** Anchored rocky slope, Dynamic calculation method, Newmark displacement method, Stability safety factor, Gaussian mixture model, Failure probability, Natural hazards, Civil engineering

## Abstract

The seismic deterioration effects of anchor cables and slope structural planes are often neglected in the dynamic stability analysis of anchored rocky slopes to the extent that the stability of slopes is overestimated. In this paper, a dynamic calculation method for anchored rocky slopes considering the seismic deterioration effect is established, and a stability evaluation method for anchored rocky slopes based on the Gaussian mixture model is proposed. The seismic deterioration effect on the stability of anchored rocky slopes is quantitatively analyzed with an engineering example, and the relationship between seismic intensity and the failure probability of slopes is clarified. The results show that compared with the calculation method without considering the seismic deterioration effect, the minimum safety factor and post-earthquake safety factor obtained by the proposed method in this paper are smaller. The number of seismic deteriorations of the slope is used as the number of components of the Gaussian mixture model to construct the failure probability model of the slope, which can accurately predict the failure probability of anchored rocky slopes. The research results significantly improve the accuracy of the stability calculation of anchored rocky slopes, which can be used to guide the seismic design and safety assessment of anchored rocky slopes.

## Introduction

Earthquake-induced slope instability is a major secondary geological hazard, which has the characteristics of wide distribution, large number and serious damage^1,2^. As a flexible support structure with active force, the prestressed anchor cable has good seismic performance and is widely used in slope seismic reinforcement engineering^3,4^. In the analysis of the dynamic stability of anchored rocky slopes, most scholars pay attention to the influence of seismic loads, but often neglect the deterioration effect caused by earthquakes^5^. However, under seismic loads, the slip deterioration effect and the friction attenuation effect of the structural plane and the damage effect of anchor cables will occur in the anchored rocky slope, which will lead to the reduction of the shear strength and stiffness of the structural plane and the anchoring force and stiffness of the anchor cable. Neglecting the seismic deterioration effect will result in an overestimation of the calculated value of the safety factor.

The slip deterioration effect of the structural plane is mainly manifested in the damage of the structural plane during the shear process between the sliding body and the bedrock under seismic load. Many scholars have carried out shear tests, numerical simulations and theoretical analyses on rock structural planes, and have achieved fruitful research results. Plesha et al. proposed a physical constitutive law for the behavior of geologic discontinuities with dilatancy and contact surface degradation to describe rock joints, including the effects of dilatancy, asperity surface degradation and bulking^6^. Based on the research results of Plesha, Qi proposed a method for calculating the seismic permanent displacement of rocky slopes considering the degradation of structural planes based on the traditional Newmark displacement method^7^. Zhang et al. proposed that slight changes of the surface of the structural plane during the shearing process usually lead to changes in the shear strength of the structural plane, and the shear strength of the structural plane gradually decreases with the increase of the shear displacement^8^. In addition, the variation of shear stiffness during the shearing process of the structural plane has gradually attracted the attention of researchers. Tang et al. carried out direct shear tests on artificial structural planes, and the results showed that the shear stiffness decreases and gradually approaches a constant with the increase of the shear displacement^9^. Wu et al. carried out cyclic shear tests on structural planes with different shear displacements, and found that with the increase of the number of cycles, the shear stiffness gradually decreases and eventually tends to a constant^10^. Wu et al. carried out loading and unloading tests on structural planes, and proposed a fitting formula for the functional relationship between the shear stiffness and the shear displacement of the structural plane^11^.

In addition to the slip deterioration effect, the seismic deterioration effect also includes the friction attenuation effect, which is closely related to the relative velocity of the sliding body and the bedrock during earthquakes. Wang et al. took the lead in carrying out the friction test of the sliding plane of granite and clarified the negative exponential relationship between the relative velocity and the shear strength of the structural plane^12^. Crawford et al. believed that the frictional resistance of structural planes depended on the shear velocity, and for harder rocks, the frictional resistance decreased as the shear velocity increased^13^. Atapour et al. carried out shear tests on structural planes with different shear velocities under constant normal load boundary conditions, and found that the shear strength of soft rock structural planes decreases with the increase of shear velocity^14^. Liu et al. redeveloped FLAC3D based on the functional relationship between the relative velocity and the shear strength of the structural plane, and obtained the influence factor of the relative velocity^5^. Ni and Gao fully considered the influence of relative velocity in the calculation of slope stability, and introduced the relative velocity damage coefficient as a reduction factor in the process of solving slope stability^15,16^. It can be seen that the frictional attenuation effect of relative velocity on the structural plane cannot be neglected.

In the stability analysis of anchored slopes under earthquakes, the change in axial force of the anchor cable is generally not considered. However, it has been confirmed by a large number of shaking table tests and numerical simulation studies that the axial force of the anchor cable changes with the seismic acceleration during earthquakes, and even the anchor cable fracture occurs^17–19^. Yan et al. regarded the anchor cable as a linear elastic material, and proposed a theoretical formula for calculating the dynamic safety factor of the anchored slope considering the change of the axial force of the anchor cable^20^. Jia et al. used a negative exponential function as the axial force calculation model of the anchor cable under earthquakes, and proposed the dynamic stability calculation formula for the bedding rocky slope^21^. Yan and Jia fully considered the change of the axial force of the anchor cable caused by the slip of the sliding body, but ignored the fluctuation of the axial force of the anchor cable during the whole period of the earthquake. According to Ye and Dong the axial force of the anchor cable fluctuates around the initial prestress during earthquakes^22,23^. However, none of the above studies considered the problem of anchor cable failure due to axial force overload during high-intensity earthquakes.

Nowadays, there are many methods to analyze the stability of slopes under earthquakes, including the quasi-static method^24–30^, the quasi-dynamic method^31–35^, the Newmark displacement method^36–40^, and the time history analysis method^41–44^. The essence of the quasi-static method is to equivalently convert the inertial force acting on the rock mass into the product of the seismic acceleration and the weight of the sliding body, and solve the safety factor through the limit analysis method or the limit equilibrium method, which only considers the seismic intensity and ignores the influence of the seismic dynamic parameters. The quasi-dynamic method is a further improvement of the quasi-static method, which fully considers the dynamic effect of seismic load on the slope. However, it is difficult to reflect the stability of the slope under real earthquakes because the quasi-dynamic method equates seismic waves to simple concordant waves to calculate the safety factor of the slope. The Newmark displacement method takes the yield acceleration as the criterion for determining the slope slip, and the slip displacement is obtained by integrating the slip acceleration quadratically. However, this method lacks a failure criterion for judging slope stability and is difficult to apply to practical engineering. The time history analysis method calculates the inertial force in each time step through the dynamic balance equation and obtains the safety factor at each time, finally forming a time history curve of the safety factor. This method can effectively reflect the real stability change of the slope under earthquakes, but cannot provide an evaluation index for the overall stability of the slope. Therefore, it has been proposed to use the normal distribution function to fit the probability distribution of the safety factor time history of the slope to obtain the failure probability of the slope^45^. However, the normal distribution function cannot accurately fit the distribution of the slope failure probability considering the effect of seismic deterioration.

In this paper, the calculation formula of the dynamic safety factor of anchored rocky slopes considering the seismic deterioration effect is derived, and the stability evaluation method of anchored rocky slopes based on the Gaussian mixture model is established. In addition, the effects of seismic deterioration on the dynamic response and the dynamic safety factor of anchored rocky slopes are also thoroughly investigated, the feasibility of the Gaussian mixture model for stability evaluation of anchored rocky slopes is verified, and the effects of seismic intensity on the stability of anchored rocky slopes are summarized.

## Seismic deterioration effect

The safety factor of anchored rocky slopes is the ratio of anti-sliding force to sliding force. Under the action of an earthquake, the anti-sliding force provided by the anchor cable and the structural plane may be reduced to varying degrees due to the seismic deterioration of the slope. The main reasons are as follows.

1. Slip deterioration effect of structural planes^11,16^: According to the Newmark displacement method, the slope slips when the seismic acceleration is greater than the yield acceleration of the slope. Slope slippage will inevitably cause abrasion of the structural plane, thereby reducing the shear strength and stiffness of the structural plane.
2. Frictional attenuation effect of structural planes^5,15^: Under the seismic cyclic load, the sliding body and the bedrock will inevitably produce relative velocity during earthquakes. The relative velocity leads to a decrease in the friction coefficient of the structural plane, which reduces the strength of the structural plane.
3. Damage effect of prestressed anchor cables^17,20^: During earthquakes, damage effects may occur in anchor cables subjected to the tensile action of the sliding body, resulting in different degrees of deterioration of the axial force and stiffness of anchor cables.

### Slip deterioration effect of structural planes

**Figure 1:**
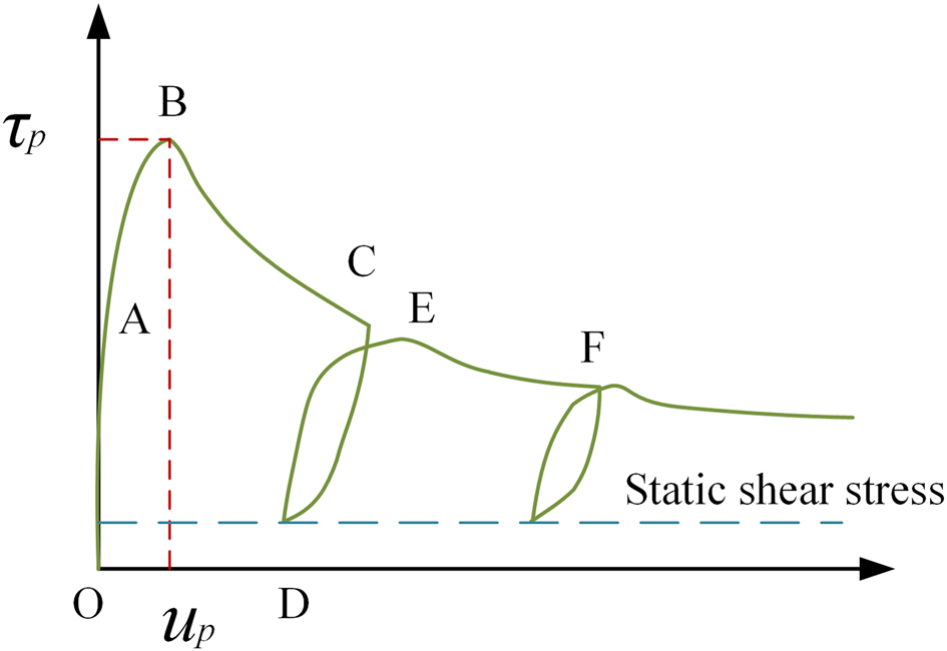
Tangential stress path diagram of structural plane.

The slope slip process triggered by the earthquake can be regarded as the structural plane shear process under displacement-controlled loading, and the shear displacement is the slip distance of the slope, which can be obtained by the Newmark displacement method. Figure 1 is the shear stress path diagram of the structural plane under multiple slips of the slope. The stress path of the first slope slip process is O → A → B → C → D. Where OA is the rising section of shear stress, and point B is the peak point of shear stress. When the slip displacement exceeds the peak displacement *u*_*b*_, the shear stress begins to enter the BC descending section. Finally, the stress curve returns to point D when the displacement loading ends. On the whole, the shear stress path of the structural plane from the beginning to the end of the slip of the slope is basically the same as the stress path of the tangential loading–unloading of the structural plane. That is, there is an elastic deformation of the structural plane at the beginning of loading, and the tangential stress increases linearly with the displacement. As the loading displacement increases further, the shear stress decreases beyond the peak shear stress. At the end of the displacement loading, part of the elastic deformation of the structural plane is restored, and the shear stress is reduced to the static shear stress (shear stresses under static forces such as prestress and gravity).

The shear stress–shear displacement curves during the first slip of the slope usually show a significant peak. As the slip distance increases, the structural plane is continuously worn, and the shear stress–shear displacement curve of the structural plane no longer shows significant peaks during subsequent slips.

The peak shear stress of the structural plane during the first slip of the slope is:

1 $$ \tau_{p} = \sigma_{n} {\text{tan}}\left( {\alpha_{k} + \varphi_{b} } \right) $$

where $\varphi_{b}$ is the fundamental friction angle of a smooth structural plane, $\alpha_{k}$ is the undulation angle of the structural plane, and $\sigma_{n}$ is the normal stress exerted on the structural plane.

The peak shear stress of the structural plane that has been severely worn after several slips is:

2 $$ \tau_{p} = \sigma_{n} {\text{tan}}\varphi_{b} $$

The degree of deterioration of the slope structural plane is closely related to the slope slip displacement. Gao et al. ^16^ proposed a method to fit the shear displacement-degradation coefficient with a negative exponential function, and the calculation formula of the shear strength degradation coefficient of the structural plane can be obtained by normalizing the shear displacement:

3 $$ \eta \left( \varepsilon \right) = Ae^{ - B\varepsilon } + C $$

4 $$ \varepsilon = \sum {u_{rel} /L} $$

where $A$, $B$ and $C$ are the coefficients to be determined, $u_{rel}$ is the one slip displacement calculated by the Newmark displacement method, and $L$ is the length of the structural plane.

According to Plesha’s study ^6^, the calculation formula of the undulating angle degradation of the structural plane under cyclic shear action is as follows:

5 $$ \alpha_{k} = \alpha_{k0} \cdot e^{{\left( { - 0.114JRC\left( {\sigma_{n} /JCS} \right)W^{P} } \right)}} $$

where $\alpha_{k0}$ is the initial undulation angle of the structural plane, $JRC$ is the roughness coefficient of the structural plane, $JCS$ is the uniaxial compressive strength of rock, and $W^{P}$ is the plastic work.

The formula for calculating the degradation of the undulation angle of the structural plane is organized as the product of the degradation coefficient of the undulation angle and the initial undulation angle.

6 $$ \alpha_{k} = \eta \left( {W^{P} } \right)\alpha_{k0} $$

The degradation coefficient is a function related to the plastic work, and its expression is as follows:

7 $$ \eta \left( {W^{P} } \right) = e^{{\left( { - 0.114JRC\left( {\sigma_{n} /JCS} \right)W^{P} } \right)}} $$

According to Eq. (7), it is known that the degradation coefficient of the undulation angle of the structural plane has a negative exponential relationship with $JRC\left( {\sigma_{n} /JCS} \right)$. Therefore, Eq. (3) can be further modified to obtain the Eq. (8) for the degradation coefficient of the undulation angle of the structural plane.

8 $$ \eta \left( \varepsilon \right) = Ae^{{ - B\varepsilon \left( {\sigma_{n} /JCS} \right)JRC}} + C $$

As a result, a degradation equation for the undulation angle of the structural plane related to the shear displacement can be obtained as follows:

9 $$ \alpha_{k} = \eta \left( \varepsilon \right)\alpha_{k0} $$

According to the theoretical derivation of Dong ^46^, the correspondence between the undulation angle and the dilatancy angle of the structural plane is obtained as follows:

10 $$ \alpha_{k0} = 2\alpha_{d0} $$

Barton ^47^ conducted experiments on eight different rough structural planes and proposed a conversion formula for the dilatancy angle to the roughness coefficient of the structural plane.

11 $$ \alpha_{d0} = \frac{JRC}{2}\log_{10} \left( {\frac{JCS}{{\sigma_{n} }}} \right) $$

Equation (12) can be obtained by substituting Eqs. (9)–(11) into Eq. (1):

12 $$ \tau = \sigma_{n} \cdot \tan \left( {\varphi_{b} + \eta \left( \varepsilon \right)JRC\log_{10} \left( {\frac{JCS}{{\sigma_{n} }}} \right)} \right) $$

Therefore, the degradation coefficient of the undulation angle can be considered as the degradation coefficient of the roughness of the structural plane.

According to Wu's study ^11^, it was found that the degradation law of tangential stiffness is similar to that of tangential strength of the structural plane. Therefore, the degradation formula of tangential stiffness of the structural plane can be expressed by negative exponential function as follows:

13 $$ k_{s} = k_{s0} \left( {De^{{ - E\varepsilon \left( {\sigma_{n} /\sigma_{c} } \right)JRC}} + F} \right) $$

Where $k_{s0}$ is the initial tangential stiffness of the structural plane, and $D$, $E$ and $F$ are the coefficients to be determined.

### Slip deterioration effect of structural planes

Wang ^12^ clarified that the dynamic friction coefficient is composed of the starting friction coefficient and the velocity-dependent function linearity through the sliding plane friction test of granite, and the expression is as follows:

14 $$ f_{k} = f_{s} \cdot f_{\mu } \left( {v_{{\text{s}}} } \right) $$

where $f_{s}$ is the starting friction coefficient, $v_{{\text{s}}}$ is the relative velocity of the upper block relative to the bedrock, and $f_{\mu } \left( {v_{{\text{s}}} } \right)$ is a function that decreases as $\left| {v_{{\text{s}}} } \right|$ increases.

Based on Wang’s research results, Ni ^15^ proposed the hypothesis that the peak shear strength of the structural plane has a negative exponential function relationship with the relative velocity, and gave the calculation formula of the relative velocity damage coefficient.

15 $$ \gamma \left( t \right) = \gamma_{r} - \left( {1 - \gamma_{r} } \right)e^{{ - a\left| {v\left( t \right)} \right|}} $$

where $\gamma_{r}$ is the convergence value of the relative velocity attenuation, and $a$ is a coefficient to be determined. Based on the calculation of Liu ^5^, Ni ^15^ and Gao ^16^, it’s specified that $\gamma_{r} = 0.9$ and $a = 25$ in this paper.

Therefore, considering the influence of the frictional attenuation effect and slip deterioration effect on the shear strength of the structural plane, the formula for calculating the shear strength of the structural plane can be obtained as follows:

16 $$ \tau = \gamma \left( t \right) \cdot \sigma_{n} {\text{tan}}\left( {\varphi_{b} + \eta \left( \varepsilon \right)JRC\log_{10} \left( {\frac{JCS}{{\sigma_{n} }}} \right)} \right) $$

Liu ^5^ transformed the formula for calculating the shear strength of the structural plane and deduced the formula for calculating the equivalent friction angle related to the undulation angle as follows:

17 $$ \varphi = {\text{arctan}}\left[ {\gamma \left( t \right) \cdot {\text{tan}}\left( {\varphi_{b} + \alpha_{k} } \right)} \right] $$

Substituting Eqs. (9)–(11) into Eq. (17), the formula for calculating the equivalent friction angle with respect to the roughness coefficient of the structural plane is obtained as follows:

18 $$ \varphi = {\text{arctan}}\left[ {\gamma \left( t \right) \cdot {\text{tan}}\left( {\varphi_{b} + \eta \left( \varepsilon \right)JRC\log_{10} \left( {\frac{JCS}{{\sigma_{n} }}} \right)} \right)} \right] $$

### Damage effect of prestressed anchor cables

**Figure 2:**
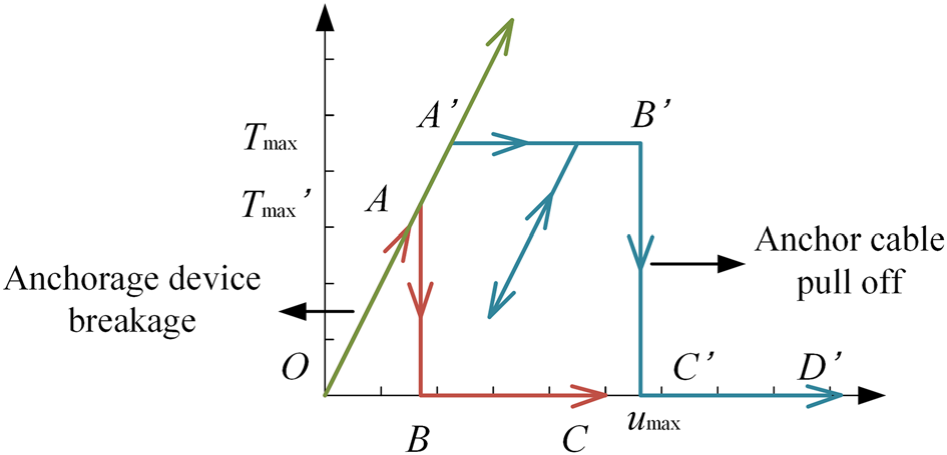
Calculation model of anchor cable.

Anchor cables are flexible support structures that can only withstand tensile forces but cannot resist bending moments and shear forces. The failure modes of anchor cables are generally divided into brittle failures such as anchor head cracking and anchor pier collapse, and ductile failures such as anchor cable breakage. Among them, the maximum axial force is usually used as the failure index for brittle failure mode, while the elongation is used as the failure index for ductile failure due to the obvious yield stage of the anchor cable ^17^. Based on the above considerations, the calculation model of the anchor cable for the brittle failure mode and the ductile failure mode is proposed in this paper. Figure 2 shows the p-s curve of the calculation model of the anchor cable. The brittle failure process of the anchor cable is composed of an elastic stage (AB) and a failure stage (BC), and the ductile failure process is composed of an elastic stage (Aʹ Bʹ), a plastic stage (Bʹ Cʹ) and a failure stage (Cʹ Dʹ). When the anchor cable is in the elastic phase (OA or OAʹ), the stiffness of the anchor cable is the initial stiffness, and the static axial force of the anchor cable is related to the slip displacement of the sliding body. If the slope does not slip, the static axial force is the prestress of the anchor cable. If the slope slips, the static axial force is the sum of the prestress of the anchor cable and the increase in the axial force of the anchor cable caused by the slope sliding. The dynamic axial force of the anchor cable is the sum of the static axial force and the increment of the axial force of the anchor cable caused by the earthquake. When the anchor cable is in the plastic stage (AʹBʹ), the stiffness of the anchor cable is 0, and the static axial force and dynamic axial force of the anchor cable are the axial force when the anchor cable yields. When the anchor cable is in the failure stage (BC or CʹDʹ), the stiffness of the anchor cable is 0, and the static axial force and dynamic axial force of the anchor cable are 0.

The calculation model of the anchor cable for the brittle failure mode is:

19 $$ \left\{ {\begin{array}{*{20}l} {T_{p} = T_{0} + k_{f} u_{p} ,\;k_{f} = EA/l,\;T_{d} = k_{f} u_{f} ,\;T_{d} < T_{\max } ^{\prime}} \hfill \\ {T_{p} = 0,\;k_{f} = 0,\;T_{d} = 0,\;T_{d} \ge T_{\max } ^{\prime}} \hfill \\ \end{array} } \right. $$

where $T_{p}$ is the static axial force of the anchor cable, $T_{0}$ is the initial prestress of the anchor cable, $T_{d}$ is the dynamic axial force of the anchor cable, $T_{\max } ^{\prime}$ is the maximum axial force of the brittle failure of the anchor cable, $k_{f}$ is the stiffness of the anchor cable, $u_{p}$ is the total slip displacement of the sliding body, $u_{f}$ is the total stretch of the anchor cable in the axial direction under the earthquake, and $l$ is the length of free section of the anchor cable.

The calculation model of the anchor cable for the ductile failure mode is:

20 $$ \left\{ {\begin{array}{*{20}l} {T_{p} = T_{0} + k_{f} u_{p} ,\;k_{f} = EA/l,\;T_{d} = k_{f} u_{f} ,\;T_{d} < T_{\max } \& u_{f} < u_{\max } } \hfill \\ {T_{p} = T_{\max } ,\;k_{f} = 0,\;T_{d} = T_{\max } ,\;T_{d} \ge T_{\max } \& u_{f} < u_{\max } } \hfill \\ {T_{p} = 0,\;k_{f} = 0,\;T_{d} = 0,\;u_{f} \ge u_{\max } } \hfill \\ \end{array} } \right. $$

where $T_{\max }$ is the yield axial force of the anchor cable, and $u_{\max }$ is the maximum stretch of the anchor cable.

The total stretch of the anchor cable in the axial direction under the earthquake is

21 $$ u_{f} = u_{p} + \left( {u_{s} + \Delta u_{sf} } \right){\text{cos}}\left( {\alpha + \theta } \right) + \left( {u_{n} + \Delta u_{nf} } \right){\text{sin}}\left( {\alpha + \theta } \right) $$

where $u_{s}$ and $u_{n}$ are the displacement response of the sliding body in *s* and *n* directions during the earthquake. $\alpha$ is the inclination of the structural plane, and $\theta$ is the angle between the anchor cable and the horizontal plane.

The stretches of the anchor cable in the *s* and *n* directions under the prestress are:

22 $$ \Delta u_{sf} = \frac{{T_{0} \cdot l}}{EA}{\text{cos}}\left( {\alpha + \theta } \right) $$

23 $$ \Delta u_{nf} = \frac{{T_{0} \times l}}{EA}{\text{sin}}\left( {\alpha + \theta } \right) $$

The total slip displacement of the sliding body is the sum of each slip displacement.

24 $$ u_{p} = \sum {u_{rel} } $$

## Slope stability evaluation

In this section, the rocky slope reinforced by prestressed anchor cables is taken as the research object, considering the seismic deterioration effect of the structural plane and anchor cables during the earthquake, the time history of the safety factor of the slope during the earthquake is solved, and the stability evaluation method of the slope is established based on the Gaussian mixture model.

### Dynamic calculation model of anchored rocky slope

The basic assumptions are inevitably made when the dynamic calculation model of anchored rocky slope is established as follows.

(1) The anchored rocky slope is simplified to a two-dimensional planar model in the calculation model; (2) The sliding body is an ideal rigid body that is homogeneous, continuous, and isotropic; (3) The influence of the self-weight of anchor cables is ignored; (4) Only the dynamic effect of horizontal seismic loads on the anchored rocky slope is considered.

**Figure 3:**
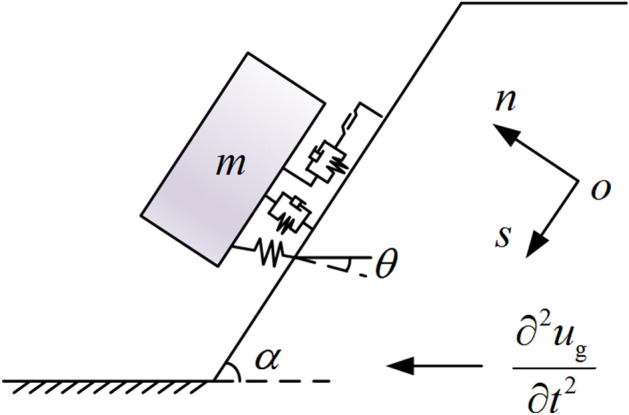
Dynamic calculation model of anchored rocky slope.

According to the dynamic calculation model of rock mass proposed by Xue ^48^, the structural plane of the slope can be regarded as a viscoelastic-plastic model. Anchor cables are usually considered as spring supports in the dynamic calculation model of the anchored slope ^22^. The dynamic calculation model of the anchored rocky slope as shown in Fig. 3 can be established.

The Newmark displacement method is used as the calculation method for the plastic slip of slopes in the dynamic calculation model of the anchored rocky slopes ^21^. The yield acceleration is the key to determining the plastic slip of the slope. The seismic force is assumed to be a horizontal static load, and the ultimate equilibrium method is used to calculate the seismic acceleration when the safety factor is 1, that is, the yield acceleration. It should be noted that with the real-time update of the deterioration parameters of the structural plane and anchor cables, the yield acceleration also changes dynamically.

**Figure 4:**
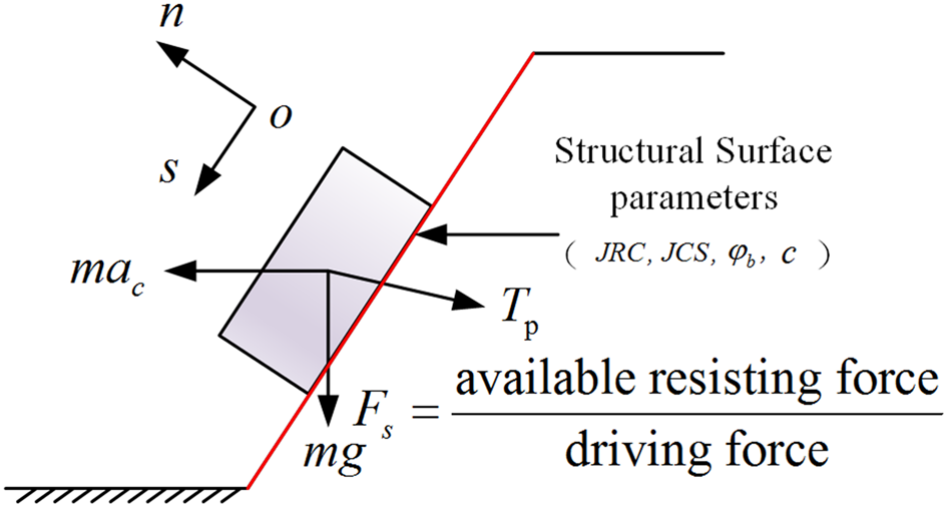
Forces acting on the sliding body of the anchored rocky slope.

Figure 4 shows the force diagram of the sliding body under earthquakes. Considering the force equilibrium condition of the sliding body, the normal force along the *n* direction is:

25 $$ N = mg{\text{cos}}\alpha - ma_{c} {\text{sin}}\alpha + T_{p} {\text{sin}}\left( {\alpha + \theta } \right) $$

where $m$ is the mass of the sliding body, and $a_{c}$ is the yield acceleration.

The sliding force along the* s* direction is:

26 $$ T = mg{\text{sin}}\alpha + ma_{c} {\text{cos}}\alpha - T_{p} {\text{cos}}\left( {\alpha + \theta } \right) $$

The anti-sliding force and sliding force of the sliding body can be obtained by analyzing the force on the sliding body. According to the limit equilibrium method, the ratio of the anti-sliding force to the sliding force is the safety factor of the slope.

27 $$ F_{s} = \frac{available \, resisting \, force}{{driving \, force}} = \frac{{c \cdot L + N \cdot {\text{tan}}\left( {\varphi_{b} + \eta \left( \varepsilon \right)JRC\log_{10} \left( {\frac{JCS}{{\sigma_{n} }}} \right)} \right)}}{T} $$

where $c$ is the cohesion of the structural plane.

The formula for calculating the safety factor of the slope can be obtained by substituting Eqs. (25) and (26) into Eq. (27).

28 $$ F_{s} = \frac{{c \cdot L + \left( {mg{\text{cos}}\alpha - ma_{c} {\text{sin}}\alpha + T_{p} {\text{sin}}\left( {\alpha + \theta } \right)} \right) \cdot {\text{tan}}\left( {\varphi_{b} + \eta \left( \varepsilon \right)JRC\log_{10} \left( {\frac{JCS}{{\sigma_{n} }}} \right)} \right)}}{{mg{\text{sin}}\alpha + ma_{c} {\text{cos}}\alpha + T_{p} {\text{cos}}\left( {\alpha + \theta } \right)}} $$

In the Newmark displacement method, the seismic acceleration with a safety factor of 1 is defined as the yield acceleration. Let $F_{s} = 1$, the expression for yield acceleration can be obtained by equation transformation as follows:

29 $$ a_{c} = \frac{{c \cdot L + mg\left( {{\text{cos}}\alpha \cdot {\text{tan}}\left( {\varphi_{b} + \eta \left( \varepsilon \right)JRC\log_{10} \left( {\frac{JCS}{{\sigma_{n} }}} \right)} \right) - {\text{sin}}\alpha } \right) + T_{p} \left( {{\text{sin}}\left( {\alpha + \theta } \right) \cdot {\text{tan}}\left( {\varphi_{b} + \eta \left( \varepsilon \right)JRC\log_{10} \left( {\frac{JCS}{{\sigma_{n} }}} \right)} \right) + {\text{cos}}\left( {\alpha + \theta } \right)} \right)}}{{m\left( {{\text{sin}}\alpha \cdot {\text{tan}}\left( {\varphi_{b} + \eta \left( \varepsilon \right)JRC\log_{10} \left( {\frac{JCS}{{\sigma_{n} }}} \right)} \right) + {\text{cos}}\alpha } \right)}} $$

### Seismic acceleration of the sliding body

The traditional Newmark displacement method takes the input seismic acceleration as the acceleration of the sliding body. However, according to Jia ^21^, it was found that seismic waves passing through a structural plane change with the stiffness of the structural plane, but neglected the deterioration effect of the stiffness and the damping effect of the structural plane. In this paper, considering the stiffness degradation effect and the structural plane damping effect, the real seismic acceleration of the sliding body is obtained by establishing the dynamic balance equation in the horizontal direction and updating the parameters of the structural plane and anchor cables in real-time.

**Figure 5:**
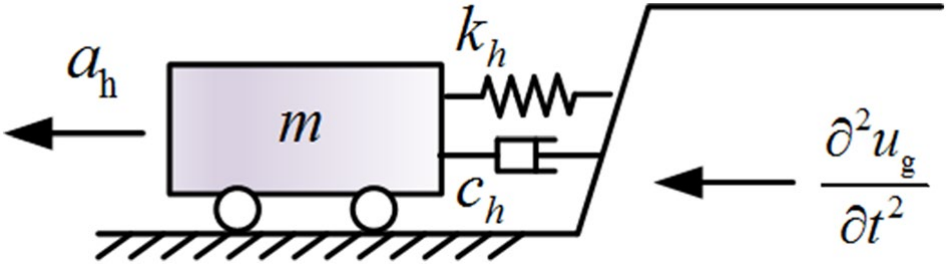
Dynamic calculation model of anchored rocky slope in the horizontal direction.

The dynamic calculation model of anchored rocky slope in the horizontal direction is shown in Fig. 5, and the dynamic equilibrium equation is established as follows:

30 $$ m\frac{{\partial^{2} u_{h} }}{{\partial t^{2} }} + c_{h} \frac{{\partial u_{h} }}{\partial t} + k_{h} \left( {u_{h} + \Delta u_{hT} } \right) = - m\frac{{\partial^{2} u_{g} }}{{\partial t^{2} }} + nT_{p} {\text{cos}}\theta $$

where $\partial^{2} u_{g} /\partial t^{2}$ is the seismic acceleration, $c_{h}$ is the damping of the structural plane in the horizontal direction, $k_{h}$ is the equivalent stiffness of the structural plane in the horizontal direction, $u_{h}$ is the displacement response in the horizontal direction, $\Delta u_{hT}$ is the static displacement in the horizontal direction under the prestress, and $n$ is the number of anchor cables.

According to the principle of equal deformation energy, the equivalent spring stiffness of the horizontal direction of the structural plane can be obtained as follows:

31 $$ k_{h} = k_{n1} {\text{sin}}^{{2}} \alpha + k_{s1} {\text{cos}}^{{2}} \alpha + k_{f} {\text{cos}}^{2} \theta $$

where $k_{n1}$ and $k_{s1}$ are the stiffness of the structural plane in the *n* and *s* directions.

For single-degree-of-freedom systems, the Duhamel integral can be used to solve the dynamics equation ^49^. The displacement and velocity at the initial moment are:

32 $$ u\left( {t = 0} \right) = 0,\;v\left( {t = 0} \right) = f\left( \tau \right)d\tau /m $$

where $f\left( \tau \right)$ is the external load at time $\tau$.

There are in the differential time interval

33 $$ dx = {\text{e}}^{{ - \xi \omega \left( {t - \tau } \right)}} \left[ {\frac{f\left( \tau \right)d\tau }{{m\omega_{D} }}{\text{sin}}\omega_{D} \left( {t - \tau } \right)} \right] $$

where $\xi$ and $\omega$ are the damping ratio and natural frequency without damping, and $\omega_{D}$ is the natural frequency with damping.

The natural frequency with damping can be obtained as follows:

34 $$ \omega_{D} = \omega \sqrt {1 - \xi^{2} } $$

The entire load history can be seen as consisting of a series of successive impulse loads, each of which produces the differential response shown in Eq. (33). The total response can be used as a superposition of all the differential responses resulting from the load time history. Integration of Eq. (33) can be obtained as follows:

35 $$ u\left( t \right) = \int_{0}^{t} {f\left( \tau \right)h\left( {t - \tau } \right)d\tau } = \frac{1}{{m\omega_{D} }}\int_{0}^{t} {f\left( \tau \right){\text{e}}^{{ - \xi \omega \left( {t - \tau } \right)}} {\text{sin}}\omega_{D} \left( {t - \tau } \right)d\tau } $$

where $h\left( {t - \tau } \right)$ is the impulse reaction, $h\left( {t - \tau } \right) = \frac{1}{{m\omega_{D} }}{\text{e}}^{{ - \xi \omega \left( {t - \tau } \right)}} {\text{sin}}\omega_{D} \left( {t - \tau } \right)$.

There is some error in the trapezoidal method for solving the definite integral, especially in the early stages of the integration. The accuracy of the recursive method for solving the Duhamel integral is higher than that of the definite integral method, so the recursive method is used in this paper to solve the dynamic response of the slope.

The recursive method assumes that the response of the slope at time *t* is known and solves for the dynamic response of the slope at time *t* + Δ*t*. When the time interval is Δ*t*, the external load corresponding to time $\tau$ is $F\left( \tau \right) = F_{t} + \left( {\frac{{F_{t + \Delta t} - F_{t} }}{\Delta t}} \right)\tau$. The dynamic response of the slope at time *t* + Δ*t* is a superposition of the following three cases: (1) Free vibration with initial condition $u\left( 0 \right) = u_{t}$ and $v\left( 0 \right) = v_{t}$; (2) Forced vibration with constant external load $F_{t}$; (3) Forced vibration with external load $F\left( \tau \right) = F_{t} + \left( {\frac{{F_{t + \Delta t} - F_{t} }}{\Delta t}} \right)\tau$. The Duhamel integrals for the above three cases are superimposed to obtain the equation for the dynamic response of the slope at time *t* + Δ*t*.

36 $$ U_{t + \Delta t} = \Gamma U_{t} + \Psi F_{t + \Delta t} $$

37 $$ F_{t} = - ma_{{\text{g}}} \left( t \right) $$

38 $$ U_{t} = \left[ {\begin{array}{*{20}c} {u_{t} } \\ {v_{t} } \\ {a_{t} } \\ \end{array} } \right] $$

39 $$ \Psi = \left[ {\begin{array}{*{20}l} {A_{4} } \hfill \\ {B_{4} } \hfill \\ {1/m - 2\xi \omega A_{4} - \omega B_{4} } \hfill \\ \end{array} } \right] $$

40 $$ \Gamma = \left[ {\begin{array}{*{20}c} {A_{1} + kA_{3} } & {A_{2} + cA_{3} } & {mA_{3} } \\ {B_{1} + kB_{3} } & {B_{2} + cB_{3} } & {mB_{3} } \\ { - \omega^{2} \left( {A_{1} + kA_{3} } \right) - 2\xi \omega \left( {B_{1} + kB_{3} } \right)} & { - \omega^{2} \left( {A_{2} + cA_{3} } \right) - 2\xi \omega \left( {B_{2} + cB_{3} } \right)} & { - \omega^{2} mA_{3} - 2\xi \omega mB_{3} } \\ \end{array} } \right] $$

41 $$ A_{1} = e^{ - \xi \omega \Delta t} \left[ {{\text{cos}}\omega_{D} \Delta t + \frac{\xi }{{\sqrt {1 - \xi^{2} } }}{\text{sin}}\omega_{D} \Delta t} \right] $$

42 $$ A_{2} = e^{ - \xi \omega \Delta t} \left[ {\frac{1}{{\omega_{D} }}{\text{sin}}\omega_{D} \Delta t} \right] $$

43 $$ A_{3} = \frac{1}{k}\left\{ {\frac{2\xi }{{\omega \Delta t}} + e^{ - \xi \omega \Delta t} \left[ { - \left( {1 + \frac{2\xi }{{\omega \Delta t}}} \right){\text{cos}}\omega_{D} \Delta t + \left( {\frac{{1 - 2\xi^{2} }}{{\omega_{D} \Delta t}} - \frac{\xi }{{\sqrt {1 - \xi^{2} } }}} \right){\text{sin}}\omega_{D} \Delta t} \right]} \right\} $$

44 $$ A_{4} = \frac{1}{k}\left\{ {1 - \frac{2\xi }{{\omega \Delta t}} + e^{ - \xi \omega \Delta t} \left[ {\frac{2\xi }{{\omega \Delta t}}{\text{cos}}\omega_{D} \Delta t + \left( {\frac{{2\xi^{2} - 1}}{{\omega_{D} \Delta t}}} \right){\text{sin}}\omega_{D} \Delta t} \right]} \right\} $$

45 $$ B_{1} = e^{ - \xi \omega \Delta t} \left[ { - \frac{\omega }{{\sqrt {1 - \xi^{2} } }}{\text{sin}}\omega_{D} \Delta t} \right] $$

46 $$ B_{2} = e^{ - \xi \omega \Delta t} \left[ {{\text{cos}}\omega_{D} \Delta t - \frac{\xi }{{\sqrt {1 - \xi^{2} } }}{\text{sin}}\omega_{D} \Delta t} \right] $$

47 $$ B_{3} = \frac{1}{k}\left\{ { - \frac{1}{\Delta t} + e^{ - \xi \omega \Delta t} \left[ {\frac{1}{\Delta t}{\text{cos}}\omega_{D} \Delta t + \left( {\frac{\omega }{{\sqrt {1 - \xi^{2} } }} + \frac{\xi }{{\Delta t\sqrt {1 - \xi^{2} } }}} \right){\text{sin}}\omega_{D} \Delta t} \right]} \right\} $$

48 $$ B_{4} = \frac{1}{k\Delta t}\left\{ {1 - e^{ - \xi \omega \Delta t} \left[ {{\text{cos}}\omega_{D} \Delta t + \frac{\xi }{{\sqrt {1 - \xi^{2} } }}{\text{sin}}\omega_{D} \Delta t} \right]} \right\} $$

where $c$ is the damping of the structural plane, and $k$ is the equivalent stiffness of the structural plane.

The seismic acceleration of the sliding body at any time *t* can be obtained by iterative calculation of Eq. (36).

### The time history of the safety factor

**Figure 6:**
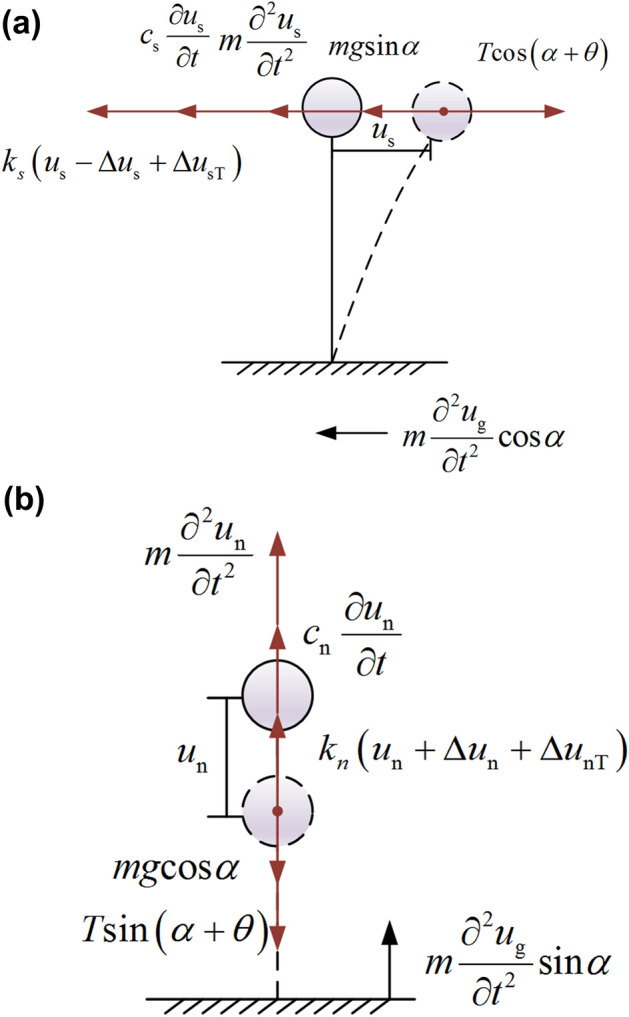
Simplified model of dynamic calculation model of anchored rocky slope (**a**) s direction (**b**) n direction.

When the seismic acceleration in the horizontal direction of the sliding body is less than the yield acceleration, the tangential plastic element is not triggered, and only the spring and damping play a role. The dynamic model of the anchored rocky slope is simplified by the centralized mass method ^50^, and the dynamic response of the slope is decomposed in the s and n directions, as shown in Fig. 6.

The dynamic balance equations for the anchored rocky slope in the *s* and *n* directions are established as follows:

49 $$ m\frac{{\partial^{2} u_{s} }}{{\partial t^{2} }} + c_{s} \frac{{\partial u_{s} }}{\partial t} + k_{s} \left( {u_{s} - \Delta u_{s} + \Delta u_{sT} } \right) = - m\frac{{\partial^{2} u_{g} }}{{\partial t^{2} }}{\text{cos}}\alpha - mg{\text{sin}}\alpha + nT_{p} {\text{cos}}\left( {\alpha + \theta } \right) $$

50 $$ m\frac{{\partial^{2} u_{n} }}{{\partial t^{2} }} + c_{n} \frac{{\partial u_{n} }}{\partial t} + k_{n} \left( {u_{n} + \Delta u_{n} + \Delta u_{nT} } \right) = - m\frac{{\partial^{2} u_{g} }}{{\partial t^{2} }}{\text{sin}}\alpha + mg{\text{cos}}\alpha + nT_{p} {\text{sin}}\left( {\alpha + \theta } \right) $$

where $c_{s}$ and $c_{n}$ are the damping of the structural plane in the *s* and *n* directions, $k_{s}$ and $k_{n}$ are the equivalent stiffness of the structural plane in the *s* and *n* directions, $\Delta u_{s}$ and $u_{n}$ are the static displacements in the *s* and *n* directions under the weight of the sliding body, $\Delta u_{sT}$ and $\Delta u_{nT}$ are the static displacements in the *s* and *n* directions under the prestress.

The equivalent stiffness of the structural plane in the* s* and *n* directions can be obtained from the principle of equal deformation energy as follows:

51 $$ k_{s} = k_{s1} + k_{f} {\text{cos}}^{{2}} \left( {\alpha + \theta } \right) $$

52 $$ k_{n} = k_{n1} + k_{f} s{\text{in}}^{{2}} \left( {\alpha + \theta } \right) $$

The tangential and normal forces acting on the structural plane during the earthquake can be obtained as follows:

53 $$ F_{\parallel } = - c_{s} \frac{{\partial u_{s} }}{\partial t} - k_{s} \left( {u_{s} - \Delta u_{s} + \Delta u_{sT} } \right) $$

54 $$ F_{ \bot } = c_{n} \frac{{\partial u_{n} }}{\partial t} + k_{n} \left( {u_{n} { + }\Delta u_{n} + \Delta u_{nT} } \right) $$

The seismic acceleration, velocity and displacement responses of the sliding body in the *s* and *n* directions at any *t* time can be obtained by iterative calculation of Eq. (36). Substituting the velocity response in the *s* direction at any time* t* into Eq. (15) can obtain the corresponding relative velocity damage coefficient. Substituting the displacement response at any time *t* into Eq. (19) or Eq. (20) can obtain the corresponding dynamic axial force and stiffness of the anchor cable. Substituting the displacement response at any time *t* into Eqs. (53) and (54), the corresponding shear force and normal force acting on the structural plane can be further obtained.

When the seismic acceleration in the horizontal direction of the sliding body is greater than the yield acceleration, the tangential plastic element of the structural plane is triggered, and the sliding body slides tangentially down along the structural plane with a slip acceleration.

The slip acceleration at time *t* can be defined as:

55 $$ a_{rel} \left( t \right) = \left( {a_{h} \left( t \right) - a_{c} \left( t \right)} \right)\left( {{\text{cos}}\alpha + {\text{sin}}\alpha \cdot {\text{tan}}\left( {\varphi_{b} + \eta \left( \varepsilon \right)JRC\log_{10} \left( {\frac{JCS}{{\sigma_{n} }}} \right)} \right)} \right) $$

The slip acceleration at time *t* can be integrated to obtain the slip velocity of the sliding body at time* t.*

56 $$ v_{rel} \left( t \right) = v_{rel} \left( {t - \Delta t} \right) + \int_{t - \Delta t}^{t} {a_{rel} \left( t \right)} dt $$

By integrating the slip velocity, the slip displacement of the sliding body at time *t* can be obtained as follows:

57 $$ u_{rel} \left( t \right) = \int_{t - \Delta t}^{t} {v_{rel} \left( t \right)} dt $$

The slip displacement of the sliding body is substituted into Eq. (12) and Eq. (13) respectively to obtain the tangential strength and stiffness of the structural plane after slip deterioration. The slip displacement of the sliding body is substituted into Eq. (19) or Eq. (20) respectively to obtain the dynamic axial force and the stiffness of the anchor cable after damage.

Based on the limit equilibrium method ^51–53^, the dynamic safety factor at time* t* can be obtained by substituting the tangential force and normal force acting on the structural plane at any time *t*, the degradation coefficient of the roughness at time *t*, and the relative velocity damage coefficient at time* t* into Eq. (58).

58 $$ Fos\left( t \right) = \frac{{c \cdot L + \gamma \left( t \right) \cdot F_{ \bot } \left( t \right) \cdot {\text{tan}}\left( {\varphi_{b} + \eta \left( \varepsilon \right)JRC\log_{10} \left( {\frac{JCS}{{\sigma_{n} }}} \right)} \right)}}{{F_{\parallel } \left( t \right)}} $$

### Failure probability of anchored rocky slopes

Seismic loads are usually considered as random variables of time, so the dynamic safety factor of anchored rocky slopes can also be considered as a random function of time, and the stability of slopes during earthquakes can be evaluated by the probability evaluation method ^5^. According to the central limit theorem, it is theoretically proved that the distribution of the dynamic safety factor of the slope obeys normal probability law ^45^. However, as the seismic deterioration effect of the slope gradually appears, the nonlinearity of the Quantile-Quantum curve of the dynamic safety factor becomes more and more significant, indicating that the probability distribution of the dynamic safety factor deviates from the normal distribution. At this point, the Gaussian mixture model can better express the complex probability distribution of the dynamic safety factor.

Gaussian mixture models are combinations of two or more Gaussian probability density functions and are very popular in density estimation and clustering ^54–56^. Although in some cases the number of components in a Gaussian mixture model may be unlimited, in general, the number of components is limited to a finite ^57^. The probability density function of a Gaussian mixture model can be written in the form of sum:

59 $$ p\left( x \right) = \sum\limits_{{k = 1}}^{K} {\pi _{k} } {\mathcal{N}}\left( {x\left| {\mu _{k} ,\sigma _{k} } \right.} \right),\;\sum\limits_{{k = 1}}^{K} {\pi _{k} }  = 1,\;0 \le \sum\limits_{{k = 1}}^{K} {\pi _{k} }  \le 1 $$

60 $$ \pi  \equiv \left\{ {\begin{array}{*{20}l}    {\pi _{1} ,} \hfill & {\pi _{2} ,} \hfill & {\pi _{3} ,} \hfill &  \ldots  \hfill & {\pi _{k} } \hfill  \\   \end{array} } \right\} $$

61 $$ \mu \equiv \left\{ {\begin{array}{*{20}c} {\mu_{1} ,} & {\begin{array}{*{20}c} {\mu_{2} ,} & {\mu_{3} } \\ \end{array} ,} & {\ldots} & {\mu_{k} } \\ \end{array} } \right\} $$

62 $$ \sigma \equiv \left\{ {\begin{array}{*{20}c} {\sigma_{1} ,} & {\begin{array}{*{20}c} {\sigma_{2} ,} & {\sigma_{3} } \\ \end{array} ,} & {\ldots} & {\sigma_{k} } \\ \end{array} } \right\} $$

where $\pi$ is a parameter vector including the weight coefficients of the components of the Gaussian mixture model, $\mu$ is a parameter vector including the means of the components of the Gaussian mixture model, $\sigma$ is a parameter vector including the variances of the components of the Gaussian mixture model, and $k$ is the number of components of the Gaussian mixture model.

The seismic deterioration effect of the anchored rocky slope will cause shifts in the median of the safety factor time history of the slope, and the median value of the safety factor time history is closely related to the expectation of the components of the Gaussian mixture model. Therefore, when the Gaussian mixture model is used as the probability density function of the dynamic safety factor of the slope, the number of seismic deteriorations can be used as the number of components of the Gaussian mixture model. It should be noted that the number of seismic deteriorations is the sum of the number of slips of the sliding body and the number of anchor cable failures.

Maximum likelihood estimation is an efficient method to obtain probability distribution parameters by maximizing the logarithm of the likelihood function. The EM algorithm is commonly used to calculate maximum likelihood estimates in the presence of latent variables or missing data and can be used to determine the parameters of a Gaussian mixture model. The EM algorithm is an efficient iterative method where each iteration consists of two processes^57^:

1. E-step: Solve the expectation $p\left( {Z\left| {X,} \right.\theta^{\left( t \right)} } \right)$.
2. M-step: Solve the maximum and calculate the model parameter $\theta^{{\left( {t{ + 1}} \right)}}$ for a new iteration.

In step E, given the current estimate of the parameter $\theta^{\left( t \right)}$, the conditional distribution of *Z* is determined as the proportional height of the normal density weighted by $\pi$:

63 $$ \gamma \left( {z_{nk} } \right) = p\left( {Z\left| {X,} \right.\theta^{\left( t \right)} } \right) = \frac{{\pi_{k}^{\left( t \right)} {\mathcal{N}}\left( {x_{n} \left| {\mu_{k}^{\left( t \right)} ,\sigma_{k}^{\left( t \right)} } \right.} \right)}}{{\sum\nolimits_{j = 1}^{K} {\pi_{n}^{\left( t \right)} {\mathcal{N}}\left( {x_{j} \left| {\mu_{j}^{\left( t \right)} ,\sigma_{j}^{\left( t \right)} } \right.} \right)} }} $$

where $\theta_{k} = \left( {\pi_{k} ,\mu_{k} ,\sigma_{k} } \right)$ is a parameter vector including the weight, mean and variance of the Gaussian component of ${\mathcal{N}\ominus }_{k}$.

In step M, the next estimate $\theta^{{\left( {t{ + 1}} \right)}}$ can be determined by maximizing the conditional expectation Q in step E, and calculated by Eq. (64).

64 $$ \theta^{{\left( {t + 1} \right)}} = {\text{arg max}}{\mathcal{Q}}\left( {\theta ,\theta^{\left( t \right)} } \right) $$

The expectation *Q* in Eq. (64) can be constructed as follows:

65 $$ {\mathcal{Q}}\left( {\theta ,\theta^{\left( t \right)} } \right) = \sum\limits_{Z} {p\left( {Z\left| {X,} \right.\theta^{\left( t \right)} } \right)} {\text{ln}}p\left( {X,Z\left| \theta \right.} \right) = \sum\limits_{{z_{1} }} \cdots \sum\limits_{{z_{N} }} {\left\{ {\sum\limits_{n = 1}^{N} {{\text{ln}}p\left( {x_{n} ,z_{n} \left| \theta \right.} \right)\prod\limits_{n = 1}^{N} {p\left( {z_{n} \left| {x_{n} } \right.,\theta^{\left( t \right)} } \right)} } } \right\}} $$

The $\pi_{k}$, $\mu_{k}$ and $\sigma_{k}$ are separate linear terms, so they can be maximized independently. According to $\frac{{\partial {\mathcal{Q}}}}{{\partial \pi_{k} }} = 0$, $\frac{{\partial {\mathcal{Q}}}}{{\partial \mu_{k} }} = 0$, $\frac{{\partial {\mathcal{Q}}}}{{\partial \sigma_{k} }} = 0$ and s.t $\sum\nolimits_{k = 1}^{K} {\pi_{k} } = 1$, the next estimates can be obtained:

66 $$ \pi_{k}^{{\left( {t{ + 1}} \right)}} = \frac{{N_{k} }}{N} $$

67 $$ \mu_{k}^{{\left( {t + 1} \right)}} = \frac{1}{{N_{k} }}\sum\limits_{n = 1}^{N} {\gamma \left( {z_{nk} } \right)} x_{n} $$

68 $$ \sigma_{k}^{{\left( {t + 1} \right)}} = \frac{1}{{N_{k} }}\sum\limits_{n = 1}^{N} {\gamma \left( {z_{nk} } \right)} \left( {x_{n} - \mu_{k}^{{\left( {t + 1} \right)}} } \right)\left( {x_{n} - \mu_{k}^{{\left( {t + 1} \right)}} } \right)^{T} $$

69 $$ N_{k} = \sum\limits_{n = 1}^{N} {\gamma \left( {z_{nk} } \right)} $$

The Technical Code for Building Slope Engineering (GB50330-2013) stipulates the minimum value of the stability safety factor of the slope of each safety grade under seismic conditions, and the required minimum values of the safety factor of the grade I, II and III slopes are 1.15, 1.1 and 1.05, respectively. In this evaluation method, the minimum safety factor value required by the code is used as the evaluation index of slope stability, and the probability of being less than the required minimum safety factor value is defined as the failure probability of the slope.

### Stability evaluation steps for anchored rocky slopes

Based on the time history analysis method and probability analysis method, a dynamic stability analysis method of anchor rock slope considering the seismic degradation effect is proposed. The calculation steps are as follows.

1. The quasi-static method is used to analyze the force of the sliding body, and the yield acceleration of the sliding body is obtained by Eq. (29).
2. The dynamic equation of the slope in the horizontal direction is established by Eq. (30). The seismic acceleration of the sliding body at the current time step is obtained by the Duhamel integral recursion method. By comparing the relationship between the yield acceleration and the seismic acceleration of the sliding body, it is determined whether the plastic slip of the slope is triggered.
3. When $a_{c} > a_{h}$, the sliding body does not slip. The dynamic equations of the slope in the s and n directions are established by Eqs. (49) and (50). The acceleration, velocity and displacement of the sliding body at the current time step are obtained by the Duhamel integral recursion method. Substituting the velocity into Eq. (15) to obtain the relative velocity damage coefficient of the sliding body. Substituting the displacement into Eqs. (19) or (20) to obtain the static axial force, dynamic axial force and stiffness of the anchor cable. When $a_{c} < a_{h}$, the slip displacement of the sliding body at the current time step is calculated by Eq. (57). Substituting the slip displacement into Eqs. (8) and (13) to obtain the degradation coefficient of the roughness and degradation stiffness of the structural plane. Substituting the slip displacement into Eqs. (19) or (20) to obtain the static axial force, dynamic axial force and stiffness of the anchor cable.
4. The results of the calculation in step (3) are used to update the current calculation parameters, and the updated calculation parameters are re-substituted into steps (1) to (3) to carry out the dynamic calculation for the next time step. This cycle is repeated until the end of the calculation.
5. The time history of the safety factor of the slope is obtained by substituting the tangential force and normal force of the structural plane, the relative velocity damage coefficient of the sliding body and the degradation coefficient of the roughness at each time step into Eq. (58).
6. The number of seismic deteriorations of the anchored rocky slope is counted, and the number of seismic deteriorations is used as the number of components of the Gaussian mixture model to establish the probability density function of the Gaussian mixture model.
7. The appropriate slope stability evaluation index is selected, and the slope failure probability corresponding to this index is obtained from the cumulative distribution function of the anchored rocky slope.

## The case study

### The basic parameters of the slope

**Figure 7:**
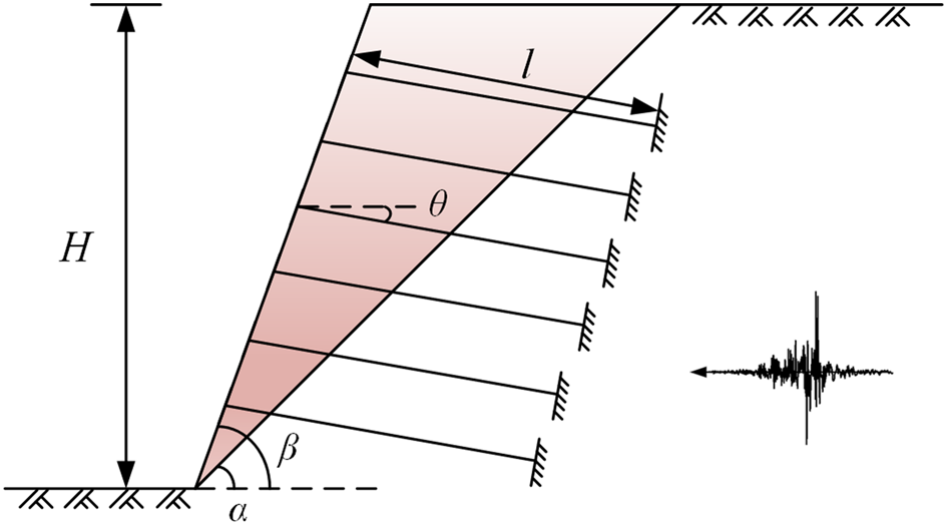
Schematic diagram of the anchored rocky slope.

**Figure 8:**
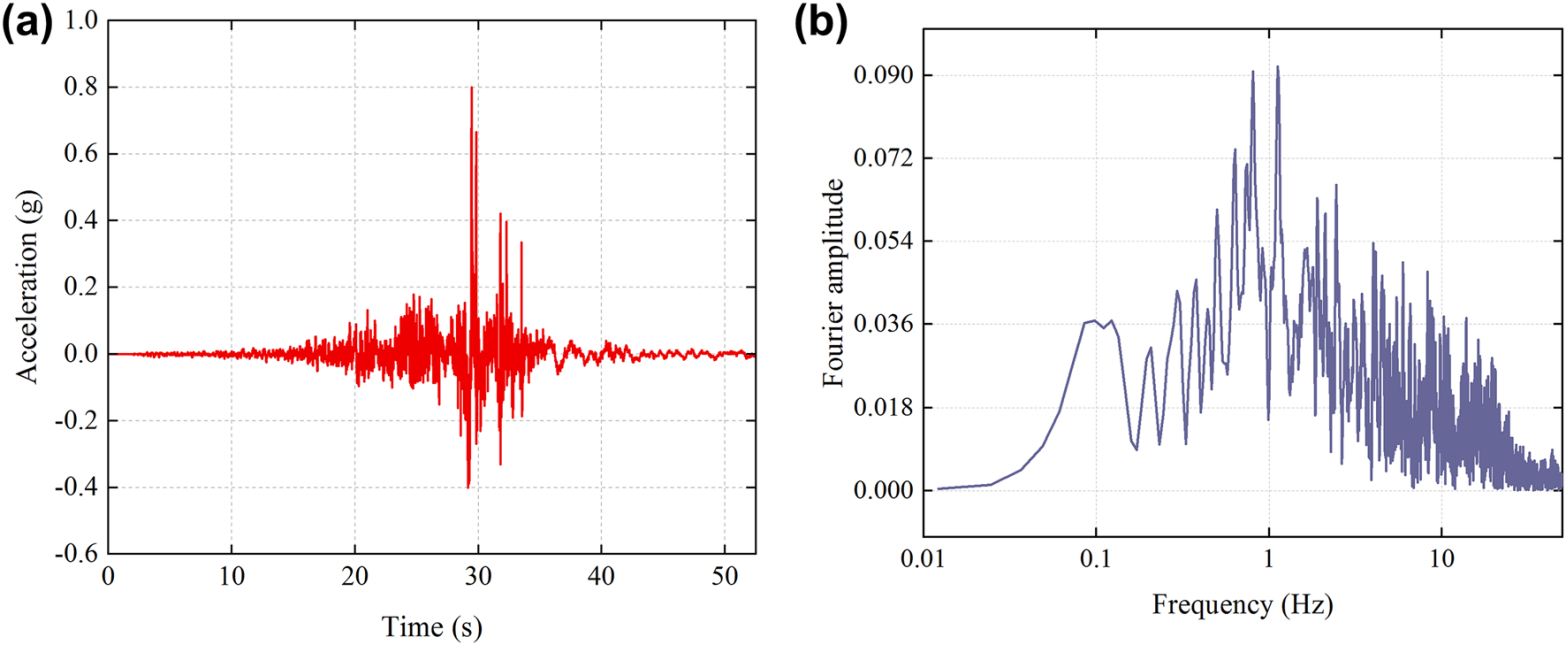
ChiChi wave and the corresponding spectrum (**a**) Seismic acceleration time history (**b**) Spectrum.

In this paper, a specific engineering example is selected to quantitatively analyze the dynamic response and stability of anchored rocky slopes. As shown in Fig. 7, the slope height *H* = 14 m, the slope inclination angle* β* = 70°, and the structural plane inclination angle *α* = 45°. The rock mass density *ρ* = 2700 kg/m^3^, the structural plane initial roughness *JRC* = 12.3, the basic friction angle *φ*_*b*_ = 25°, the cohesion *c* = 35 MPa, the rock strength *JCS* = 120 MPa, the damping ratio *ξ* = 0.15, the normal stiffness *k*_*n*_ = 3 MPa, and the tangential stiffness *k*_*s*_ = 1 MPa. The slope is arranged with six rows of prestressed anchor cables from top to bottom. The anchor cable type is 2φ15.2. The length of the free section *l* = 6 m, the anchor cable inclination angle *θ* = 10°, the initial prestress *T*_*f*_ = 100 kN, and the maximum bearing tension force *T*_*max*_ʹ = 136.5 kN. The coefficients to be determined for the roughness and tangential stiffness degradation formulas for the structural plane obtained by least squares fitting are listed in Table 1. In this case, the input seismic wave is the ChiChi wave with a PGA of 0.8 g in the horizontal direction, and the time history curve and spectrum of the seismic wave after filtering and baseline correction are shown in Fig. 8.

**Table 1:** The pending coefficient in the roughness and tangential stiffness degradation formulas.

Roughness degradation formula $\eta \left( \varepsilon \right) = Ae^{{ - B\varepsilon \left( {\sigma_{n} /JCS} \right)JRC}} + C$		Tangential stiffness degradation formula $k_{s} = k_{s0} \left( {De^{{ - E\varepsilon \left( {\sigma_{n} /\sigma_{c} } \right)JRC}} + F} \right)$	
Pending coefficient	Value	Pending coefficient	Value
A	0.8025	D	11.7648
B	1874.48	E	6007.95
C	0.6371	F	8.0340

### Analysis of the results

#### Influence of seismic deterioration effect

**Figure 9:**
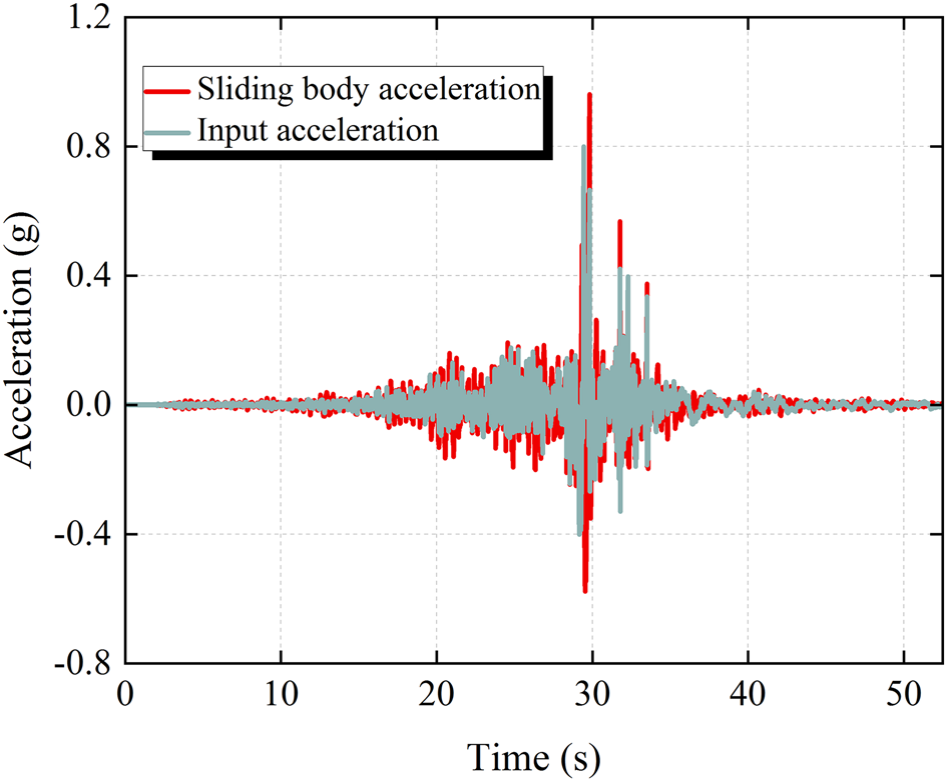
Acceleration time history of the sliding body and input acceleration time history.

Figure 9 shows the comparison of the seismic acceleration time history of the sliding body with the input seismic acceleration time history. From Fig. 9, it can be seen that the waveform of the input seismic acceleration time history is basically similar to that of the seismic acceleration time history of the sliding body, but the fluctuation amplitude of the seismic acceleration time history of the sliding body is larger than that of the input seismic acceleration time history, which is 0.96 g. The difference in the amplitude between the seismic acceleration time history of the sliding body and the input seismic acceleration time history is mainly related to the frequency of the input seismic acceleration time history and the natural frequency of the slope. Failure of the anchor cable or slippage of the sliding body reduces the equivalent stiffness of the structural plane, which in turn reduces the natural frequency of the slope. This may result in the superior frequency of the input seismic acceleration time history being closer to the natural frequency of the slope, so that the slope produces a certain resonance effect, thereby increasing the amplitude of the seismic acceleration time history of the sliding body. In addition, due to the damping effect of the structural plane, the PGA of the sliding body is delayed with respect to the input PGA.

**Figure 10:**
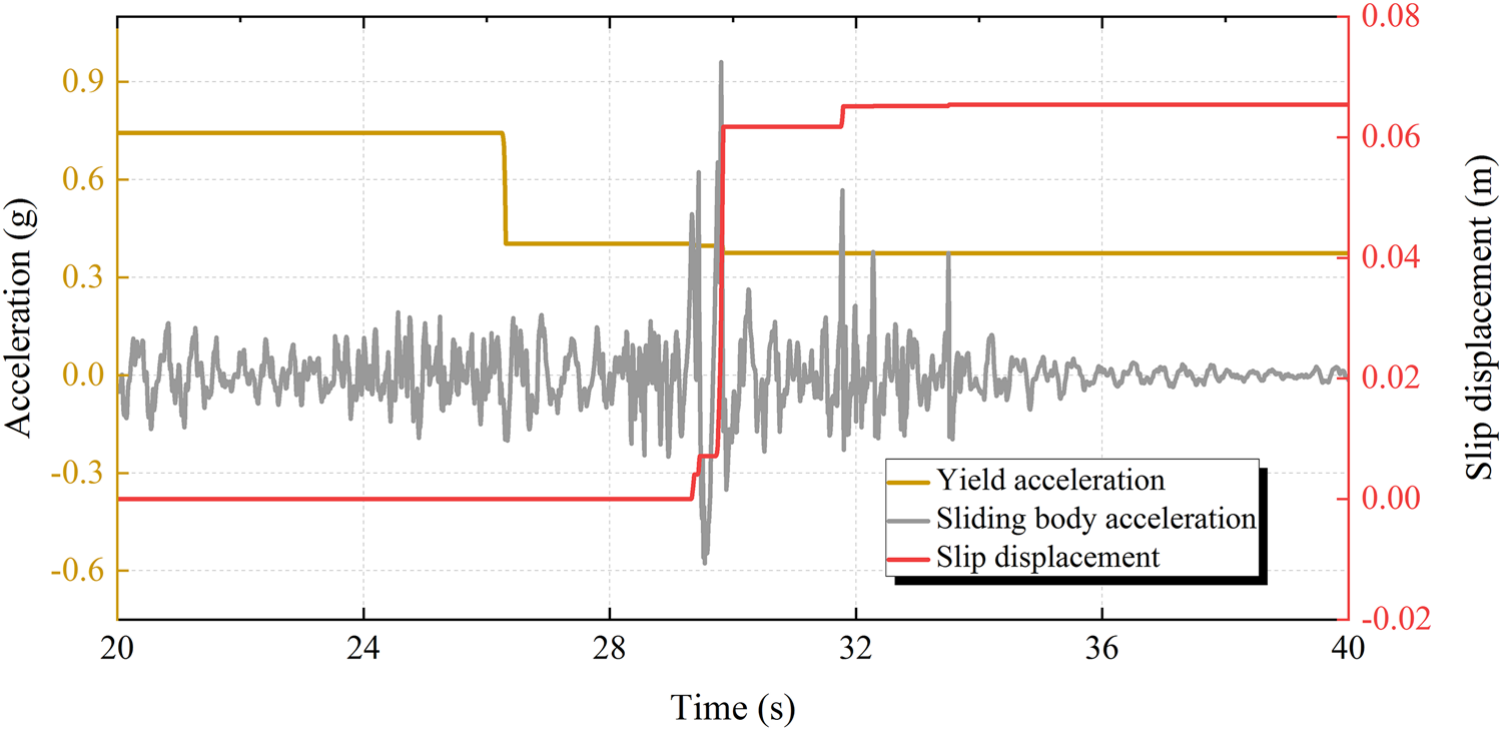
Displacement calculated using the method in this paper.

In the traditional Newmark displacement method, the yield acceleration is considered to be a constant value that does not change during the earthquake. In this paper, the slip deterioration of the structural plane and the damage of anchor cables are fully considered during the earthquake, and the yield acceleration of the sliding body is updated in real-time to obtain a more reasonable slip displacement of the sliding body. As can be seen from Fig. 10, the yield acceleration of the sliding body decreases several times in different degrees. When the yield acceleration drops abruptly for the first time, the slip displacement of the sliding body is 0, indicating that the decrease in yield acceleration is not caused by the degradation of the structural plane but by the failure of anchor cables. Then, with the multiple slips of the sliding body, the yield acceleration gradually decreases, and the final yield acceleration tends to a constant. In general, the seismic deterioration effect will reduce the yield acceleration of the sliding body, and thus increase the slip displacement of the sliding body.

**Figure 11:**
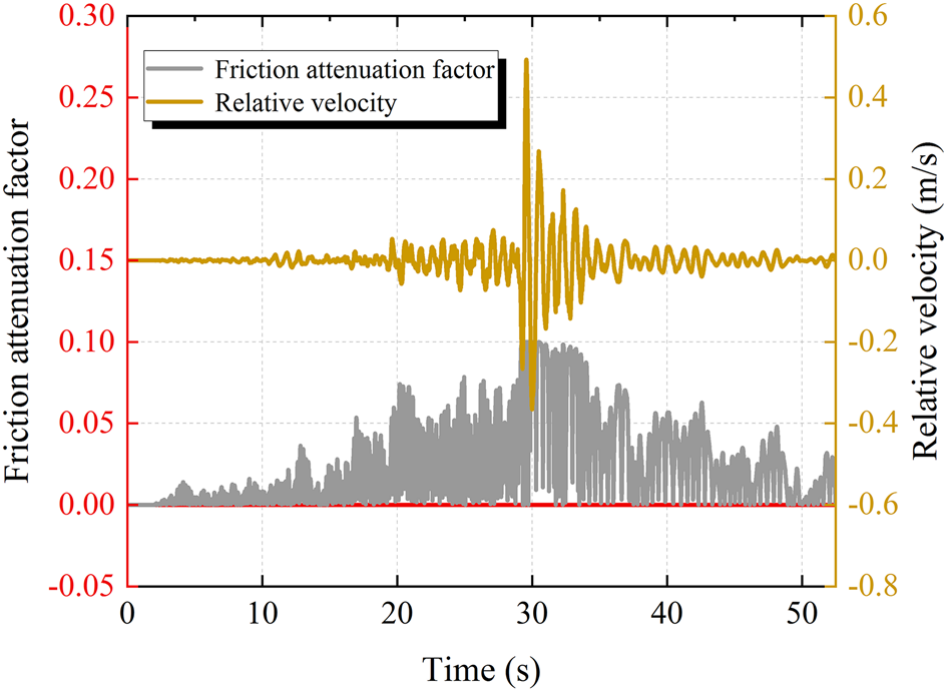
Friction attenuation factor time history.

In order to clarify the influence of the relative velocity of the sliding body on the friction attenuation effect of the structural plane, the friction attenuation factor is used in this paper to describe the degree of friction attenuation of the structural plane. It is worth noting that the friction attenuation factor and the relative velocity damage coefficient are the sum of 1. Figure 11 shows the time history of the friction attenuation factor during the earthquake. The frictional attenuation effect occurs when the relative velocity of the sliding body and the bedrock is generated under the earthquake, which will cause a temporary reduction of the shear strength of the structural plane. It can be seen from Fig. 11 that the friction attenuation factor fluctuates in the range of 0–0.10, and its magnitude is closely related to the relative velocity. With the increase of the relative velocity, the relative friction attenuation factor increases, but its growth rate decreases. In addition, the frictional attenuation effect appears during the earthquake and disappears after the earthquake, and the friction attenuation factor after the earthquake is 0. This result is consistent with the results of Liu's research ^5^ on the relative velocity damage coefficient.

**Figure 12:**
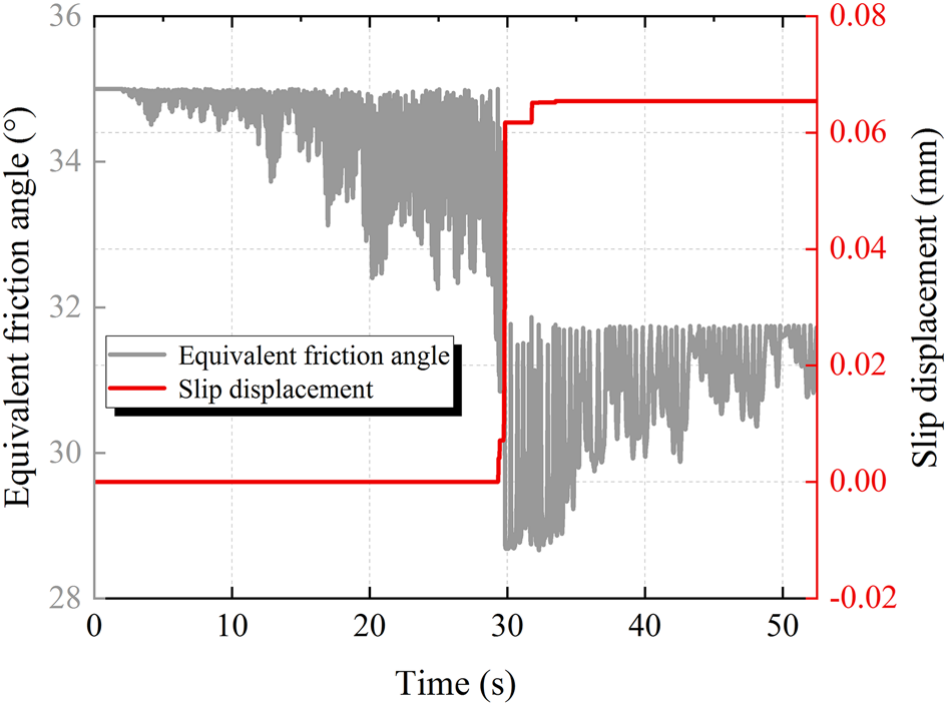
Equivalent friction angle time history.

Figure 12 shows the time history of the equivalent friction angle during the earthquake. As can be seen from Fig. 12, the equivalent friction angle time history is mainly composed of two forms, one is a recoverable temporary reduction with seismic load fluctuations, and the other is a non-recoverable permanent reduction with slip displacement. The former is caused by the frictional attenuation effect associated with the relative velocity, and the equivalent friction angle reduces non-permanently and recovers after the relative velocity disappears. The latter is caused by the slip deterioration effect associated with the slip displacement, the slip of the sliding body causes wear on the structural plane, resulting in a reduction in the roughness of the structural plane. Since the abrasion of the structural plane is permanent, the reduction of the equivalent friction angle due to the slip deterioration effect is irrecoverable, and the moment of sudden decrease of the equivalent friction angle corresponds to the moment of the slip of the sliding body.

**Figure 13:**
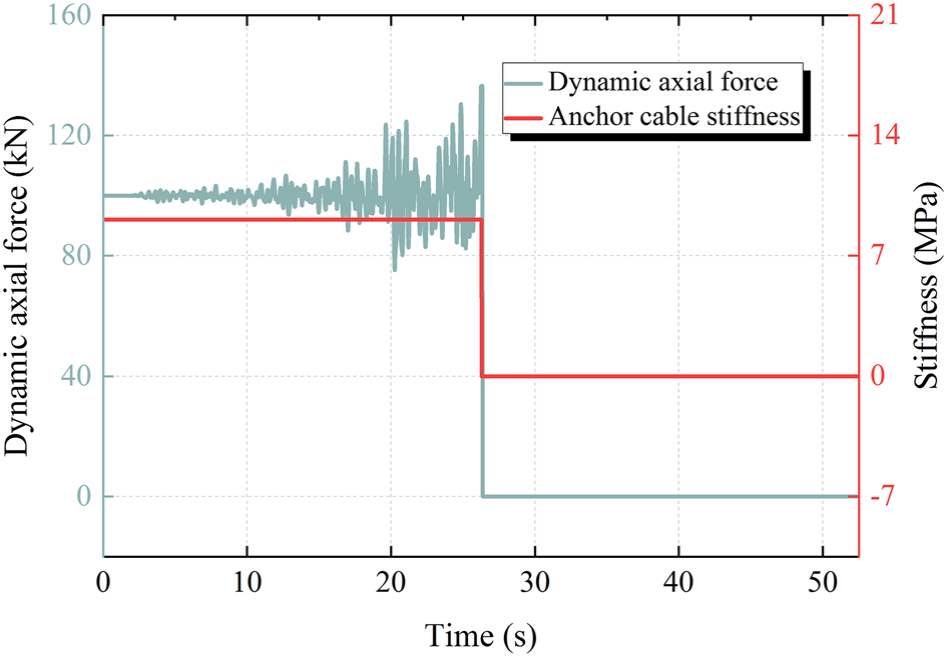
Dynamic axial force and stiffness time history of the anchor cable.

Figure 13 shows the dynamic axial force and stiffness time history of the anchor cable during the earthquake. From Fig. 13, it can be seen that the dynamic axial force of the anchor cable fluctuates within a certain range in the early stage of the earthquake. When the dynamic axial force exceeds 136.5 kN, the brittle failure of the anchor cable occurs, and the dynamic axial force decreases abruptly to 0 kN. In this process, the anchor cable does not show a significant yield stage, indicating that the failure of the anchor cable is caused by the cracking of the anchorage device or the collapse of the anchor pier rather than the fracture of the anchor cable. Since the anchor cable is always in an elastic state before failure, the stiffness remains at the initial stiffness during this process and drops to 0 after the failure of the anchor cable.

#### The results obtained by the two calculation conditions

**Figure 14:**
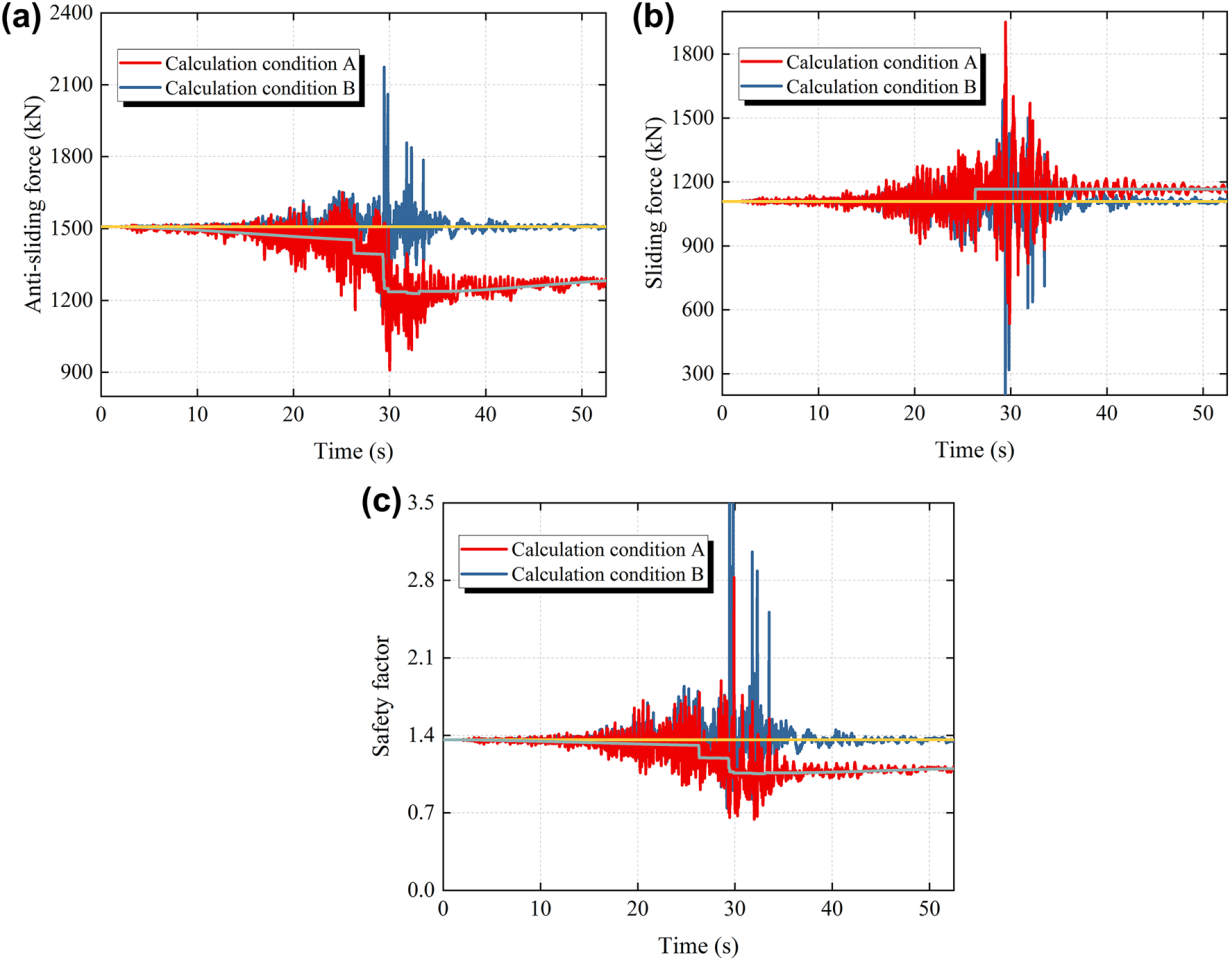
Time history of anti-sliding force, sliding force and safety factor of the slope under different calculation conditions. (**a**) Anti-sliding force time history (**b**) Sliding force time history (**c**) Safety factor time history.

In order to analyze the seismic deterioration effect on the stability of the slope, two calculation conditions are used in this paper to calculate the anti-sliding force, sliding force and safety factor of the slope, respectively. Calculation condition A: The seismic deterioration effect is considered; Calculation condition B: The seismic deterioration effect is not considered. The time history of the anti-sliding force, the sliding force and the safety factor obtained under the two calculation conditions are shown in Fig. 14a–c, respectively.

As can be seen from Fig. 14a, the trend line of the anti-sliding force time history under calculation condition A shows both an asymptotical decrease and increase, as well as precipitous drops. In the early stage of the earthquake, compared with calculation condition B, the trend line of the anti-sliding force time history under calculation condition A gradually drifts downward, while the trend line of the anti-sliding force time history slowly rises with the decrease of the earthquake intensity in the later stage of the earthquake. This is because the friction attenuation effect is related to the relative velocity, the relative velocity of the sliding body that increases first and then decreases leads to the change of the trend line in the form of an asymptotically lower and then asymptotically higher under calculation condition A. The trend line of the anti-sliding force time history under calculation condition A first decreases abruptly at 26.29 s, and then there are several sudden drops of varying degrees with the progress of the earthquake. The reason for the sudden drops in the trend line is whether the anchor cable is broken or the structural plane deteriorates, which needs to be comprehensively analyzed in combination with the sliding force time history.

From Fig. 14b, it can be found that compared with calculation condition B, the trend line of the time history of the sliding force under calculation condition A rises abruptly at 26.29 s, which is caused by the failure of anchor cables. There are two main reasons for the sudden increase in the trend line. On the one hand, the sliding body loses the normal force of anchor cables, which causes the friction force upward along the structural plane on the sliding body to decrease abruptly. On the other hand, the failure of anchor cables causes the tension upward along the structural plane on the sliding body to disappear suddenly. In addition, considering the variation trend of the time history of the anti-sliding force and the sliding force under calculation condition A in Fig. 14a,b, it can be seen that the first sudden drop of the trend line of the anti-sliding force time history is caused by the failure of anchor cables, and then the subsequent sudden drops of different degrees is caused by the slip deterioration effect of the structural plane.

As can be seen from Fig. 14c, the safety factor of the slope fluctuates to varying degrees during the earthquake. The minimum safety factor in the time history of the safety factor under calculation condition B is 0.7397, and the trend line of the time history of the safety factor remains horizontal and stabilizes at 1.3449. The minimum safety factor in the time history of the safety factor under calculation condition A is 0.6417, and the trend line of the time history of the safety factor decreases significantly and rises insignificantly, and finally stabilizes at 1.0756. Compared with calculation condition B, the minimum safety factor during the earthquake and the stable safety factor of the slope after the earthquake are reduced under calculation condition A. It can be seen that the seismic deterioration effect is considered in the calculation of the safety factor of the slope, which plays an important role in both the seismic design of the slope and the post-earthquake safety assessment of the slope.

#### The results obtained by the two calculation conditions

**Figure 15:**
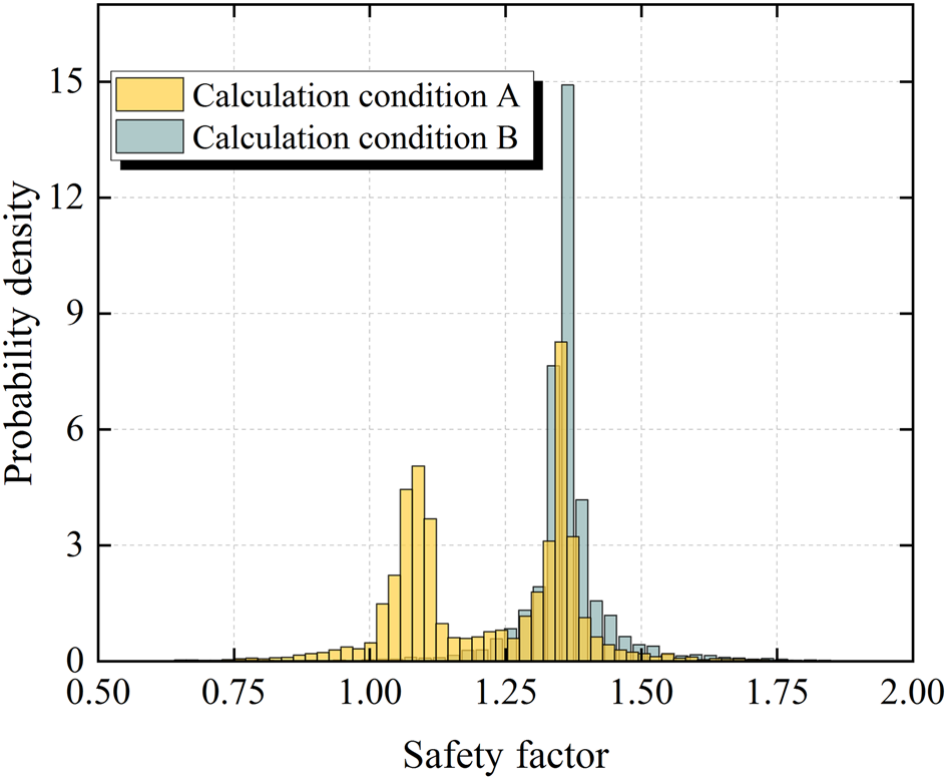
Histogram of the safety factor of the slope under different calculation conditions.

According to the time history of the safety factor under the two calculation conditions in Fig. 14c, the maximum and minimum safety factors of each curve are obtained, and the intervals of the maximum and minimum safety factors are discretized into multiple intervals with equal intervals. The safety factors of each interval are counted to obtain the number of safety factors in each discrete interval and the probability density histogram is plotted as shown in Fig. 15. As can be seen from Fig. 15, the probability density of the safety factor under calculation condition B is characterized by a unimodal distribution and obeys the normal distribution, which is consistent with Liu's research results ^45^. However, the probability density of the safety factor under calculation condition A is characterized by a bimodal or multimodal and no longer obeys the normal distribution. The Gaussian mixture model can accurately fit the probability density of multimodal distribution, so the Gaussian mixture model is used for probability density estimation of the safety factor under calculation condition A.

**Figure 16:**
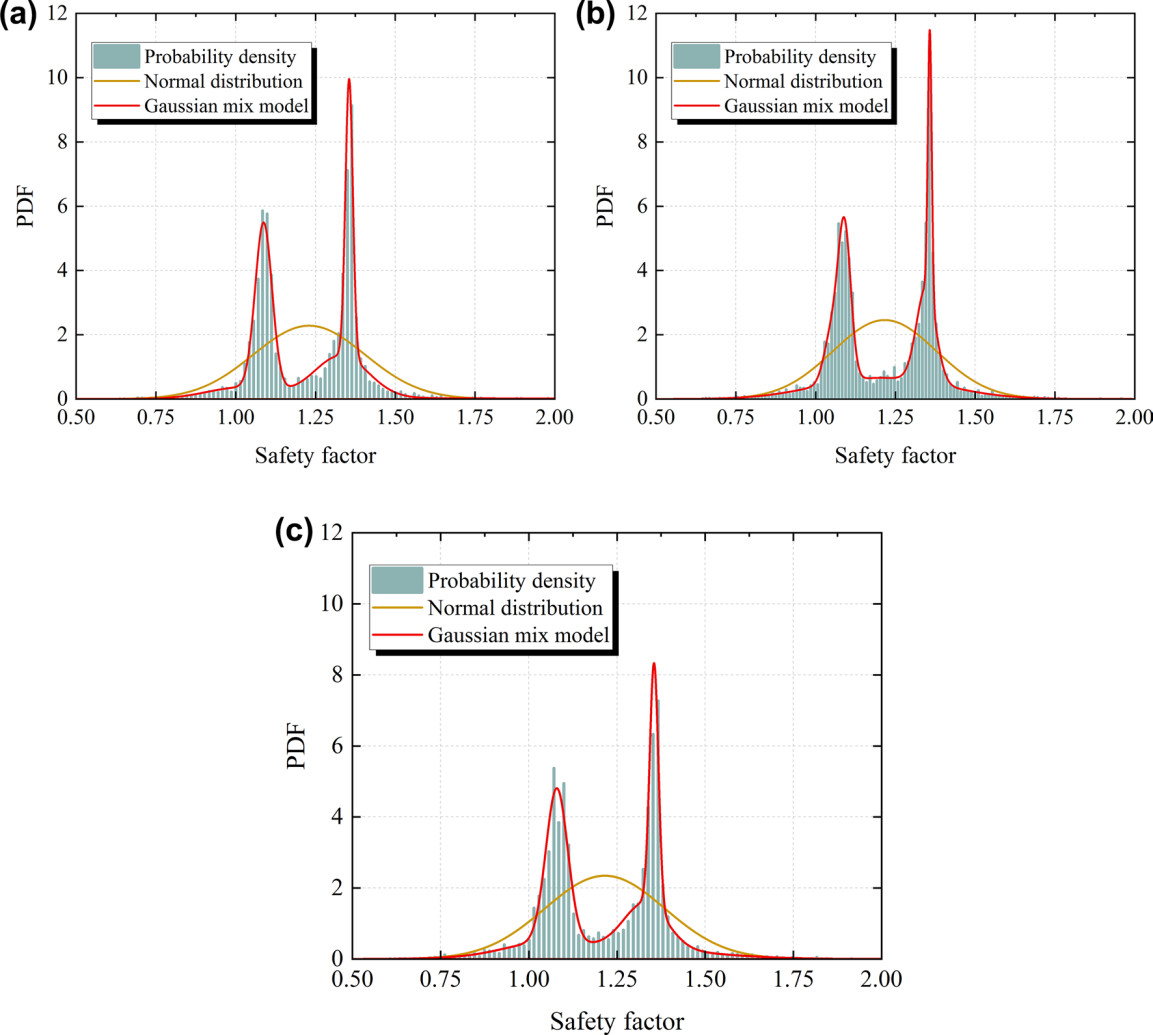
Comparison of the prediction of the probability distribution between the normal distribution model and the Gaussian mixture model (**a**) Input PGA = 0.7 g (**b**) Input PGA = 0.8 g (**c**) Input PGA = 0.9 g.

The ChiChi waves with PGAs of 0.7–0.9 g are used as the input seismic waves, and the probability distribution of the safety factor of the slope under the calculation condition A is fitted by using the normal distribution model and the Gaussian mixture model, respectively. The fitting effect is shown in Fig. 16. As can be seen from Fig. 16, the normal distribution model cannot accurately fit the probability distribution of the safety factor considering the seismic deterioration effect, while the Gaussian mixed model can accurately fit the probability distribution of the safety factor under this condition. Furthermore, the feasibility of using the number of seismic deteriorations of the slope as the number of components of the Gaussian mixture model is confirmed by accurately fitting the probability distribution of the safety factor of the slope under different seismic deterioration numbers.

**Figure 17:**
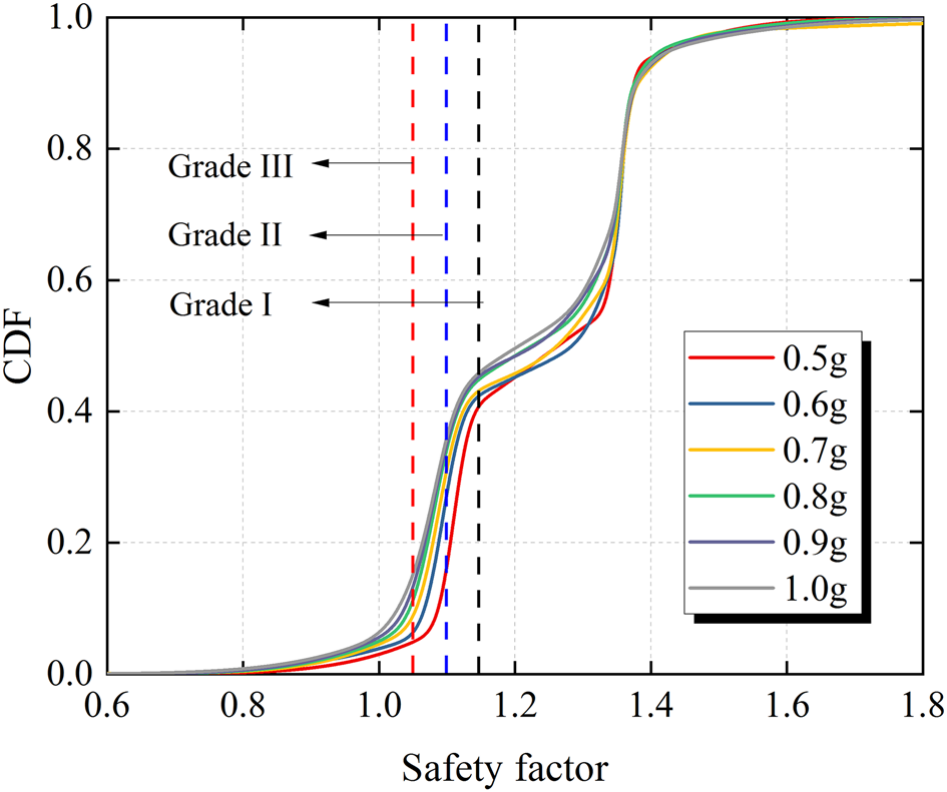
Cumulative distribution function of the safety factor under different seismic intensities.

In order to clarify the influence of seismic intensity on the failure probability of the slope, ChiChi waves with PGAs of 0.5–1.0 g are used as input seismic waves. The cumulative distribution function curves shown in Fig. 17 are plotted and the failure probability of the slope shown in Table 2 is obtained by using different safety grades as evaluation indexes. As shown in Fig. 17, the variation trend of the cumulative distribution function curve under different seismic intensities is similar, and the main difference is in the safety factor range of 0.8–1.4. In addition, with the increase of seismic intensity, the failure probability of the slope under the same failure probability evaluation index increases. It can be seen from Table 2 that the failure probability of the slope increases with the increase of the safety grade of the slope under the same seismic intensity. The number of slips increases first and then remains unchanged with the increase of seismic intensity. It should be noted that the input maximum PGA in this paper is 1.0 g, but if the seismic intensity increases further, the sliding body may slip again.

**Table 2:** Failure probability of the slope under different seismic intensities.

Input PGA (g)	Number of anchor cable breaks	Number of slope slips	Number of GMM components	Failure probability		
				Grade I	Grade II	Grade III
0.5	1	2	3	0.4120	0.1671	0.0489
0.6	1	3	4	0.4223	0.2710	0.0599
0.7	1	4	5	0.4293	0.3100	0.0837
0.8	1	6	7	0.4509	0.3442	0.1131
0.9	1	6	7	0.4538	0.3513	0.1324
1.0	1	6	7	0.4619	0.3601	0.1537

**Figure 18:**
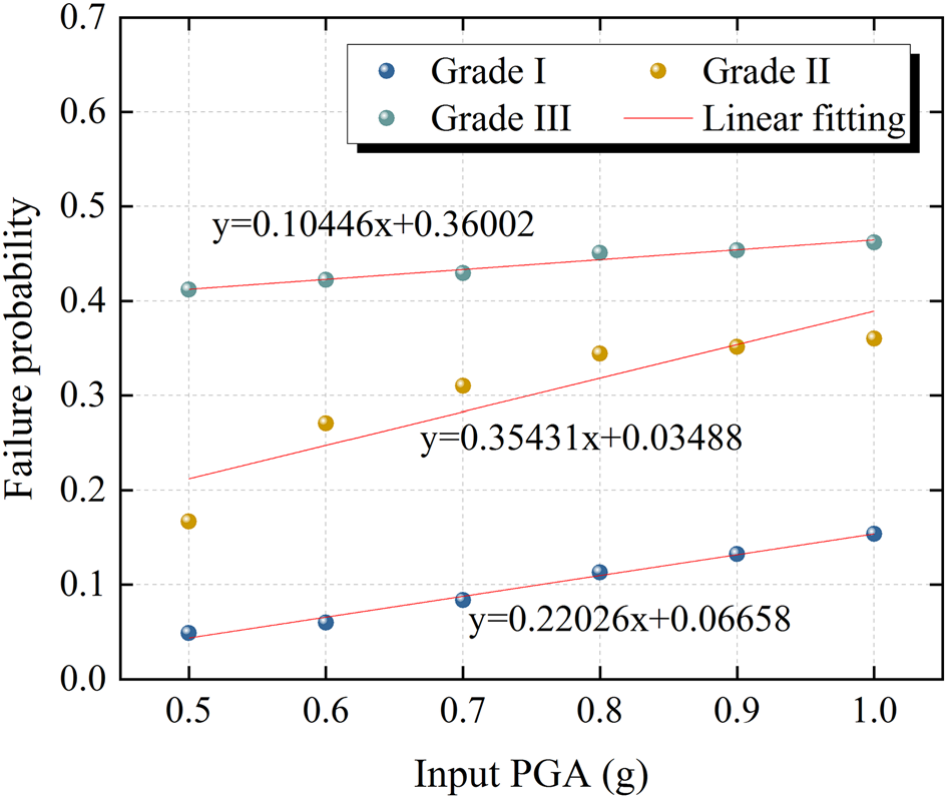
Relationship between failure probability and seismic intensity.

The fitting results of the failure probability of the slope under different seismic intensities are shown in Fig. 18. As can be seen from Fig. 18, the failure probability increases linearly with the increase of seismic intensity under the same safety grade. In this paper, only the failure probability of the slope with input PGAs of 0.5–1.0 g is calculated. In terms of the linear growth law of failure probability with seismic intensity, the failure probability of slope will further increase with the further increase of seismic intensity.

## Discussion

In the example of this paper, anchor cables undergo brittle failure before the slip of the sliding body, and the static axial force of anchor cables suddenly decreases to 0. Therefore, the damage effect of anchor cables reduces the yield acceleration of the sliding body. However, it is worth noting that the static axial force of anchor cables may increase when the sliding body slides and anchor cables do not fail. In this case, the damage effect of anchor cables will increase the yield acceleration of the sliding body thereby reducing the slip displacement of the sliding body. No matter which of the above situations occurs, the important influence of the damage effect of anchor cables on the stability of the slope cannot be ignored.

Since anchor cables in this paper have the same specifications, they failed at the same time during the earthquake. However, in the actual project, the specifications of anchor cables at different positions in the seismic design of the slope may be different, and anchor cables may be damaged one by one during the earthquake. When determining the number of components of the Gaussian mixture model for the stability evaluation, it should be noted that the number of failures of anchor cables may be many.

## Conclusion

1. The seismic deterioration effect of the structural plane can be divided into the slip deterioration effect and the friction attenuation effect. The slip deterioration effect is related to the slip displacement of the sliding body, and the roughness of the structural plane decreases exponentially with the increase of slip displacement and does not recover after the earthquake. The frictional attenuation effect is related to the relative velocity of the sliding body, which will cause temporary reductions in the shear strength of the structural plane and recovery after the earthquake.
2. Due to the resonance effect of the slope, the amplitude of the seismic acceleration time history of the sliding body may be greater than the amplitude of the input seismic acceleration time history. In addition, with the enhancement of the seismic deterioration effect, the yield acceleration of the sliding body gradually decreases. Therefore, the slip displacement calculated by the proposed method is larger than that calculated by the traditional Newmark displacement method, which is closer to the actual situation.
3. The minimum safety factor during the earthquake and the stable safety factor after the earthquake of the slope obtained by the calculation method in this paper are smaller than those of the calculation method without considering the seismic deterioration effect. It can be seen that the influence of seismic deterioration is considered in the calculation of the safety factor, which plays an important role in both the seismic design of the slope and the post-earthquake safety assessment of the slope.
4. For the seismic degradation effect of slopes, a slope stability evaluation method based on the Gaussian mixture model is proposed. The accuracy of the stability evaluation method and the feasibility of the number of seismic deteriorations as the number of components of the Gaussian mixture model are verified by an engineering example. The study on the slope failure probability under different seismic intensities shows that the slope failure probability is linearly correlated with the seismic intensity.

## Data Availability

The datasets generated during and/or analysed during the current study are available from the corresponding author on reasonable request.
