# Author Correction: Dynamic stability analysis method of anchored rocky slope considering seismic deterioration effect

**DOI:** 10.1038/s41598-024-59366-z

**Published:** 2024-04-15

**Authors:** Jinqing Jia, Xing Gao, Xiaohua Bao, Xin Xiang, Lihua Zhang, Bingxiong Tu

**Affiliations:** 1 State Key Laboratory of Coastal and Offshore Engineering, School of Civil Engineering, Dalian University of Technology, Dalian, 116024 China; 2 College of Civil and Transportation Engineering, Shenzhen University, Shenzhen, 518060 China; 3 China Three Gorges Corporation, Wuhan, 430010 China; 4 Fujian Engineering Technology Research Center for Tunnel and Underground Space, Huaqiao University, Xiamen, 361021 China

Correction to: *Scientific Reports* 10.1038/s41598-024-57413-3, published online 25 March 2024

The original version of this Article contained errors in the spelling of the authors Jinqing Jia, Xing Gao, Xiaohua Bao, Xin Xiang, Lihua Zhang & Bingxiong Tu, which were incorrectly given as Jia Jinqing, Gao Xing, Bao Xiaohua, Xiang Xin, Zhang Lihua & Tu Bingxiong respectively.

The original Article has been corrected.

